# Adhering interacting cells to two opposing coverslips allows super-resolution imaging of cell-cell interfaces

**DOI:** 10.1038/s42003-021-01960-2

**Published:** 2021-04-01

**Authors:** Julia Sajman, Yair Razvag, Shachar Schidorsky, Seon Kinrot, Kobi Hermon, Oren Yakovian, Eilon Sherman

**Affiliations:** 1 Racah Institute of Physics, The Hebrew University, Jerusalem, Israel; 2 Present Address: Graduate Program in Biophysics, Howard Hughes Medical Institute, Department of Chemistry and Chemical Biology, Department of Physics, Harvard University, Cambridge, MA USA

**Keywords:** Fluorescence imaging, Imaging the immune system

## Abstract

Cell-cell interfaces convey mechanical and chemical information in multicellular systems. Microscopy has revealed intricate structure of such interfaces, yet typically with limited resolution due to diffraction and unfavourable orthogonal orientation of the interface to the coverslip. We present a simple and robust way to align cell-cell interfaces in parallel to the coverslip by adhering the interacting cells to two opposing coverslips. We demonstrate high-quality diffraction-limited and super-resolution imaging of interfaces (immune-synapses) between fixed and live CD8^+^ T-cells and either antigen presenting cells or melanoma cells. Imaging methods include bright-field, confocal, STED, dSTORM, SOFI, SRRF and large-scale tiled images. The low background, lack of aberrations and enhanced spatial stability of our method relative to existing cell-trapping techniques allow use of these methods. We expect that the simplicity and wide-compatibility of our approach will allow its wide dissemination for super-resolving the intricate structure and molecular organization in a variety of cell-cell interfaces.

## Abstract (web summary)

Sajman et al. present a simple method to establish cell-cell interfaces for optical microscopy perpendicular to the optical axis. Widely compatible with many imaging techniques, this geometry enables to capture the cell-cell interface in the x/y plane, and allows high-quality diffraction-limited and super-resolution imaging of interfaces between interacting cells.

## Introduction

Multicellular organisms depend on cellular interactions for their structure and function. Physical interactions between cells serve for mechanical support in tissue formation, convey chemical and mechanical information and facilitate nutrient exchange between the touching cells^1^. Examples of contacts include a wide range of cellular junctions^2^, neuronal synapses^3^ and various types of immune synapses^4^. While structural contacts are relatively static and persist over days, synapses often demonstrate much faster dynamics^5^, sometimes forming within seconds^6^.

Over the past decades, optical microscopy has revealed a wealth of information regarding the structure and dynamics within such cell–cell interfaces (e.g. refs. ^6–8^). Unfortunately, the resolution of optical microscopy has been typically limited by diffraction. In a typical microscopy configuration, an *x–y* plane can be defined perpendicular to the optical axis. In this plane the resolution is limited to $dr_{x - y}\sim \lambda /2NA$, where $\lambda$ is the wavelength of imaging and *NA* is the numerical aperture of the objective. Moreover, along the optical (*z*) axis, resolution is further decreased as $dr_z = 2\lambda /\left( {NA} \right)^2$. Typical fluorescence imaging in the visible (green) range yields $dr_{x - y}\sim 170\,{\mathrm{nm}}$ and $dr_z\sim 500\,{\mathrm{nm}}$^9^. Since the interface between cell conjugates often lies perpendicular to the coverslip (i.e. along the *z*-axis), its features have been typically resolved in the worst orientation. Moreover, imaging the entire volume of cell conjugates typically requires too much time for live-cell imaging of dynamic processes using conventional confocal microscopes. Likewise, sectioning across the vertical interface is slow, because most confocal microscopes are capable of much faster horizontal scanning (e.g. video rate at 30–60 fps), relative to their vertical scanning speed. Similarly, wide-field microscopes have slow vertical imaging speeds relative to horizontal imaging. Thus, imaging the interface along a single z-plane (or a limited number of planes) could result in faster imaging and improved spatial resolution.

More recently, multiple approaches for super-resolution microscopy (SRM) have been introduced, including (but not limited to) single molecule localisation microscopy [SMLM; such as Photoactivated Localization Microscopy (PALM) or Stochastic Optical Reconstruction Microscopy (STORM)], Stimulated Emission Depletion (STED), Structured Illumination Microscopy (SIM) and their variants^10^. These methods break the diffraction limit of light, and can reach lateral resolutions of 20–30 nm. SRM has already revealed multiple nanoscale entities that play a crucial functional role in cell function. Such entities include membrane ruffles, protein clusters, protein-lipid domains, vesicles and more^11,12^. Still, for most SRM approaches, as for diffraction-limited microscopy, the resolution in the z-direction is much worse than in the *x–y* plane and its enhancement requires introducing more sophisticated components to the imaging system^13^. Thus, SRM of cell conjugates has suffered lower resolution due to the non-optimal orientation of the imaged cell–cell interface.

Compared to diffraction-limited microscopy, SRM poses more stringent requirements for imaging. For instance, SRM methods are often highly sensitive to optical aberrations and mechanical vibrations. Moreover, they often require a very low background, especially for SMLM. They further require excellent stability (movements < 20 nm/(pixel dwell time)). Otherwise, common requirements shared with diffraction-limited microscopy include compatibility with multicolor imaging using existing systems (fluorophores, excitation lasers, detectors, etc.). Deconvolution of either diffraction-limited microscopy or SRM further increases these sensitivities, as the point-spread function (PSF)^14^ is usually known and constant.

Here, we propose a simple, cost-effective and robust method for re-alignment of interfaces between cell conjugates parallel to the coverslip. Our method is based on the attachment of the two interacting cell types to opposing coverslips and then bringing them together before or during imaging, for either fixed or live-cell imaging. Spacers control the *z*-separation and the relative lateral position of the opposing coverslips. We show that our method allows most types of super-resolution imaging. Our method is further compared to alternative approaches, such as trapping cells in well arrays^15,16^ or in porous membranes.

We demonstrate the utility and performance of our method via imaging of the immune synapse (IS) between fixed and live T cells and Antigen Presenting Cells (APCs) or melanoma cells. Such ISs are an outstanding examples of cell conjugates that have critical importance in human health^17^. The specific and sensitive recognition of foreign antigens is performed by the T cell antigen receptor (TCR). Such recognition initiates a signalling cascade in the T cell, resulting in multiple effector functions^18^. The TCR signal is carefully regulated, since its over-reactivity may cause auto-immunity and graft rejection, while TCR reactivity that is too weak may cause anergy (i.e. lack of required response). In spite of the importance of TCR activation to human health, its detailed underlying mechanisms have not been fully resolved. Diffraction-limited microscopy has shown that the TCR and downstream effectors form pronounced clusters^19,20^ and that TCR triggering and Ca^++^ influx occur within seconds of first engagement of TCRs with cognate antigens^21,22^. Results from super-resolution imaging of these clusters have shown that the TCR and related signalling molecules come together in nanoclusters^23,24^ that can form dynamic and heterogeneous functional nanoscale patterns^11,24^. Importantly, unexplained localized and synchronized activation of TCRs within larger TCR clusters has been observed^25,26^. Another type of molecular patterning at the IS involves the physical separation of engaged TCRs from bulky glycoproteins in tight contacts^27^.

We show the compatibility of our approach with multiple diffraction-limited and SRM imaging modalities, including bright field, confocal and STED imaging, direct STORM (dSTORM), Super-resolution Optical Fluctuation Imaging (SOFI), and Super-Resolution Radial Fluctuations (SRRF). In addition, our technique can be used in large-scale microscopy to allow efficient scanning of multiple interacting cells. We further critically assess the pros and cons of our approach relative to current techniques for cell trapping, highlighting its superiority in stability, simplicity, wide compatibility with various microscopes and very low background. We expect that these properties of our method will allow the wide-spread use of high-quality diffraction-limited and super-resolution imaging of intercellular interfaces by enabling routine imaging using standard and advanced optical microscopes.

## Results

### A simple assay for imaging cell encounters on opposing coverslips

**Fig. 1:**
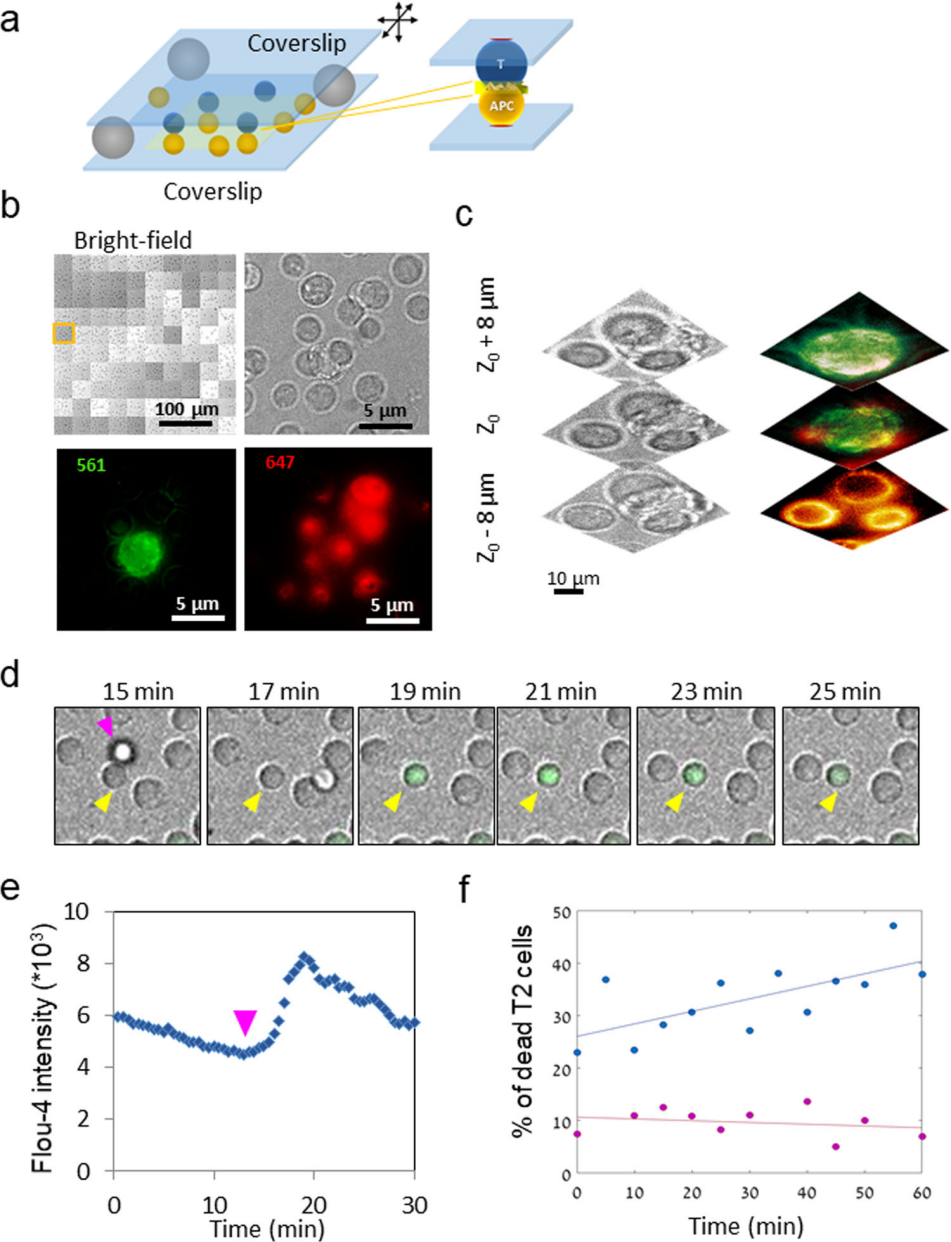
Microscopy of T/APC conjugates on opposing surfaces. **a** A schematic description of imaging T/APC conjugates on opposing coverslips. **b** Large-scale microscopy images consisting of 100 fields of view were taken of CD8^+^ T cells in contact with APC (T2 hybridoma) cells loaded with the activating peptide NY-ESO-1. A montage of large-scale bright-filed images and a single field is shown (top row). Image contrast of these images was adapted here for improved visibility of single cells. The PM of the CD8^+^ T cells was stained for αCD45 and Alexa647 (red) and the PM the T2 cells was stained using DPEE-Atto565 (green). Fluorescence images of the zoom field are shown (bottom row). Large-scale images in bright field, and in each of the fluorescence channels were routinely collected at different z-sections and are shown in Fig. S1. **c** Zoomed bright field and fluorescence images from the single field in panel **b** are shown at different heights (focal planes) relative to the cell interface. **d** Calcium imaging of Fluo-4 stained live CD8^+^ T cells (yellow arrowhead), upon encounter with the T2 cells (magenta arrowhead) loaded with the activating peptide NY-ESO-1. A representative field is shown. Yellow arrowheads show an activated cell of interest. **e** The intensity time-trajectory of Fluo-4 in the pointed CD8^+^ T cell in panel **d**. Magenta arrowhead indicates the time of the cell engagement with the T2 APC. **f** The time-dependent killing efficiency by CD8^+^ T cells of the T2 cells, with (blue) or without (magenta) loading with NY-ESO-1 peptide.

Our goal in this study was to develop a simple way to image cell–cell interfaces using SRM and large-scale microscopy. To minimize the background associated with cell-capturing layers (ref. ^16^, and as shown and discussed below), we decided to capture the cells on two opposing coverslips coated with reagents that promote cell adhesion (Fig. 1a). Such reagents may include antibodies against cell surface proteins^19^ (e.g. anti-integrins or anti-glycoproteins; αCD11a, αCD45, as shown here) or lipid bilayers with adhering molecules^28^ (e.g. ICAM1 and CD28). In our technique, each cell type is attached to a different glass surface. Prior to imaging, one cell-carrying surface is placed (upside down) on top of the second cell-carrying surface. For instance, a small coverslip can be placed on top of cells in a glass-bottom chamber. To physically separate the two surfaces, we place on the bottom surface spherical silicon beads of a desired size that matches the length of the cell–cell conjugate. Synchronization of cell–cell encounter is primarily achieved by timing the placement of the surfaces, one on top of the other. Further synchronization can be achieved by sliding one of the opposing coverslips (typically, the top coverslip in an inverted microscope) relative to the other (see Methods). The spherical beads ensure that the coverslips remain perfectly parallel during this motion.

To demonstrate this approach, we first imaged live T/APC conjugates on the opposing coverslips (Fig. 1b). In this experiment, the conjugates were CD8^+^ T cells and T2 hybridoma cells loaded with the activating peptide NY-ESO-1^29,30^. Twenty-four hours prior to the experiment, T2 hybridoma cells were treated with NY-ESO-1 peptide for MHC-I loading. Two types of glass surfaces were prepared: glass chambers (ibidi #1.5), and small glasses that fitted the opening of the chamber well (we refer to both as either coverslips or opposing surfaces). Both of the surfaces were cleaned, treated according to a previously described technique^31^ (see Methods), and subsequently coated with adhesion antibodies. Throughout this study we used the following antibodies: purified mouse anti human CD45 and mouse anti human CD11a for cells’ attachment; none of these elicit stimulation through the TCR. On the day of experiment, CD8^+^ T cells and T2 (pre-loaded with peptide) were labeled with different synthetic colors as described in each experiment. Each cell type was attached to a different glass surface. Usually T2 cells were attached to the chamber (i.e. the bottom plane), whereas CD8^+^ T cells were attached to the small glass (i.e. the upper plane). Attachment was conducted at 37 °C for 20 min. Just prior to imaging, the small glass coverslip was placed (upside down) into the chamber. We found that ~20 μm beads served well for separating the opposing surfaces and for the formation of contacts parallel to the coverslips. The CD8^+^ T cells engaged the T2 cells, the TCR and MHC-I- NY-ESO-1 peptide interaction caused T cell activation resulting in the killing of the T2 cells (see below). For fluorescence imaging of the two cells, the plasma membrane (PM) of the CD8^+^ T cells was stained for αCD45 with Alexa647 (red) and the PM of the T2 cells was stained using DPEE-Atto565 (green).

To efficiently search for cell conjugates we performed large-scale microscopy; that is we acquired a grid of 100 fields of view (FOVS; specifically, a grid of 10 × 10 FOVs, each 80 μm^2^). Imaging included both bright field and fluorescence imaging in the two spectral channels that matched the cells’ staining (Fig. 1b; also see Fig. S1). Imaging was routinely performed in three different z-sections (Figs. 1c, S1), to make sure that both the T cells and APCs are present.

Importantly, we wanted to validate that the cells could indeed create a functional synapse under the experimental conditions imposed by our assay. For that, we tested the T cell activation and killing of the APCs, carrying the cognate peptide antigen (see Methods). We first stained the CD8^+^ T cells with Fluo-4 and imaged the intracellular Ca^++^ levels in these cells upon engagement with the APCs (Fig. 1d). Indeed, we could detect robust Ca^++^ influx shortly after the engagement of the T cells and an APC (Fig. 1d, e). In a separate experiment, we further show that the CD8^+^ T cells could efficiently kill the T2 cells loaded with the cognate peptide, as compared to T2 cells that were not loaded with these peptides (Fig. 1f). See Methods for further details regarding the Ca^++^ and killing assays. Thus, we conclude that the cells demonstrated expected and productive T cell functional responses upon encounter with APCs carrying cognate antigens.

### Assessing alternatives for super-resolution imaging cell conjugates

Alternative approaches have been proposed and demonstrated for the visualization of the ISs as they form in parallel to the coverslip (Table 1). We tested the applicability of two other approaches that potentially allow visualization of multiple conjugates in wide field and in super-resolution.

**Table 1:** Comparison of approaches for cell conjugate reorientation for optimal microscopy.

Method	Opposing surfaces	PDMS well array	Pipette^c^	Optical tweezers	Acoustic trapping (with well array)	Conjugate on single coverslip (Ab-coated or GSLB)	LLSM of side-by-side conjugates	Data for opposing surfaces
Compatibility /Resolution [nm]^a^								
Wide-filed	170	>170^b^	170	170	X	170 × 500	230 (in xy) 370 (in z)	Fig. 1, S1
Confocal	170	170	?	170	170	170 × 500	NA	Figs. 2, 5
Large-scale microscopy	170	170	X	X	170	170 × 500	230 (in xy) 370 (in z	Fig. 1, S1
Live-cell imaging	170	170	V	X	170	170 × 500	230 (in xy) 370 (in z)*	Fig. 5, Movies M1,M2
SMLM (PALM)	<35	X	X	X	X	NA^e^	NA	Fig. 4B
SMLM (dSTORM)	<35	X	X	X	X	NA^e^	NA	Figs. 3, 4a, S4e, S5, S6
STED	60–70	70 × 70	X	X	V	40 × 500	NA	Fig. 2, S4a-d
SOFI	100	X^b^	X	X	X	100 × 500	NA	Fig. 4d, S7
SRRF	160	X^b^	X	?	?	?	NA	Fig. 4e, S4f
SIM^d^	100	X	X	?	?	100 × 500	NA	
Functionality								
Simplicity	V	X	X	X	X	VV	X	
Synchronization of encounter	VV	V^f^	VV	VV	V	X	X	
Robustness to different cell types	V	V	V	V	V	V	VV	Fig. 2
Yield	V	V	X	X	V	VV	V	
Alignment and stability	VV	V	VV	V	V	X No APC	VV	
Fidelity								
Physiological relevance	V^b^	For Agar	V	V Applied Forces	V Applied forces	X No APC	VV	Fig. 1
References	Newly tested	Tested here,^15,16^		^58–61^	^62,63^	Many, see review^f^	^42,43^	

*X or NA* - not applicable, or incompatible. ? - Possibly incompatible, or untested. *V* - compatible. *VV* - highly compatible.

^a^Typical resolution is estimated for ~500 nm excitation, and varies with wavelength and system.

^b^Distortions and abberations are expected.

^c^Including two pipettes or a pipette and a coverslip.

^d^Not tested here; based on aberrations from wide-field imaging.

^e^Single molecules in IS are very hard to discern due to its small apparent footprint.

^f^Requires microfluidics.

^g^Time resolution of 1.3 sec for 131 2D planes.

We first evaluated the applicability of well arrays for capturing one type of the cells (Fig. S2). In this approach, a micro-patterned well array was moulded in PDMS. The wells trap single APCs and lymphocytes. The immune synapse between the cells is thus aligned in a favourable orientation at the focal plane of a light microscope (Fig. S2a). To fabricate the micro-patterned well traps, we used patterning of a photolithographic mask. The mask was designed to accommodate a range of well sizes and spacing between wells, which subsequently helped to optimize the process of cell trapping and imaging. Next, we created a template using the mask and photolithography. The template was used to mould the wells in a curable Polydimethylsiloxane (PDMS). Polyethylene Glycol di-acrylate (PEGDA) or Agarose can serve as alternatives for moulding the wells. The well arrays were first moulded on Silicon wafers and then transferred onto the coverslips. For imaging, APCs were dropped onto the well arrays. Non-trapped cells were washed using an imaging buffer (Fig. S2b). Using this approach, we imaged single T cells (AND mouse) conjugated to APCs (B cells loaded with PCC peptide) in 15-μm well traps with confocal microscopy (Fig. S2c). T cells expressed GFP-actin (green), while B cells were marked with a non-specific cell marker (red) and both cells were stained with anti-phosphotyrosine antibodies (blue). Distinct features of T cell activation include the formation of microclusters of activated proteins (blue) and an actin ring (green). We could further image the T/APC conjugates in micro-patterned traps using a Leica TCS STED microscope (a top view with maximal intensity projection of a 3D z-stack is presented in Fig. S2d). Here, the interface was between a T cell (Jurkat E6.1; yellow) and a Raji B-cell, pulsed with a super-antigen (SEE) and serving as an APC (red). The cell-cell conjugate was imaged in cell traps (white). Strikingly, microclusters of the critical adapter protein SLP-76 (yellow dots; stained with Atto647 against pY128) could be detected at the interface with resolution down to ~70 nm. Still, trials for imaging cell conjugates through the PDMS via SMLM (PALM and dSTORM) were unsuccessful. The PDMS layer created a high background that did not allow proper localization of single emitters.

We noted that the imaging quality is sensitive to the bottom coverslip. Thus, we also tried capturing T cells (Jurkat E6.1) on an upper surface such as porous membranes with holes that match the cell size (Fig. S3a, b). While we succeeded in imaging the trapped cells using confocal microscopy (Fig. S3c), these membranes scattered enough light to effectively prohibit effective wide-field imaging through either bright field or fluorescence imaging (Fig. S3d). Notably, confocal imaging could eliminate much of the scattered light, provided that the membrane was used for capturing the upper layer of cells.

We conclude that cell capturing by either well arrays or porous membranes is suitable for confocal and STED imaging, but not for SMLM due to their high background. Hence, we decided to focus on using two opposing coverslips for the rest of this study.

### Confocal/STED imaging of cell conjugates using the opposing coverslips assay

**Fig. 2:**
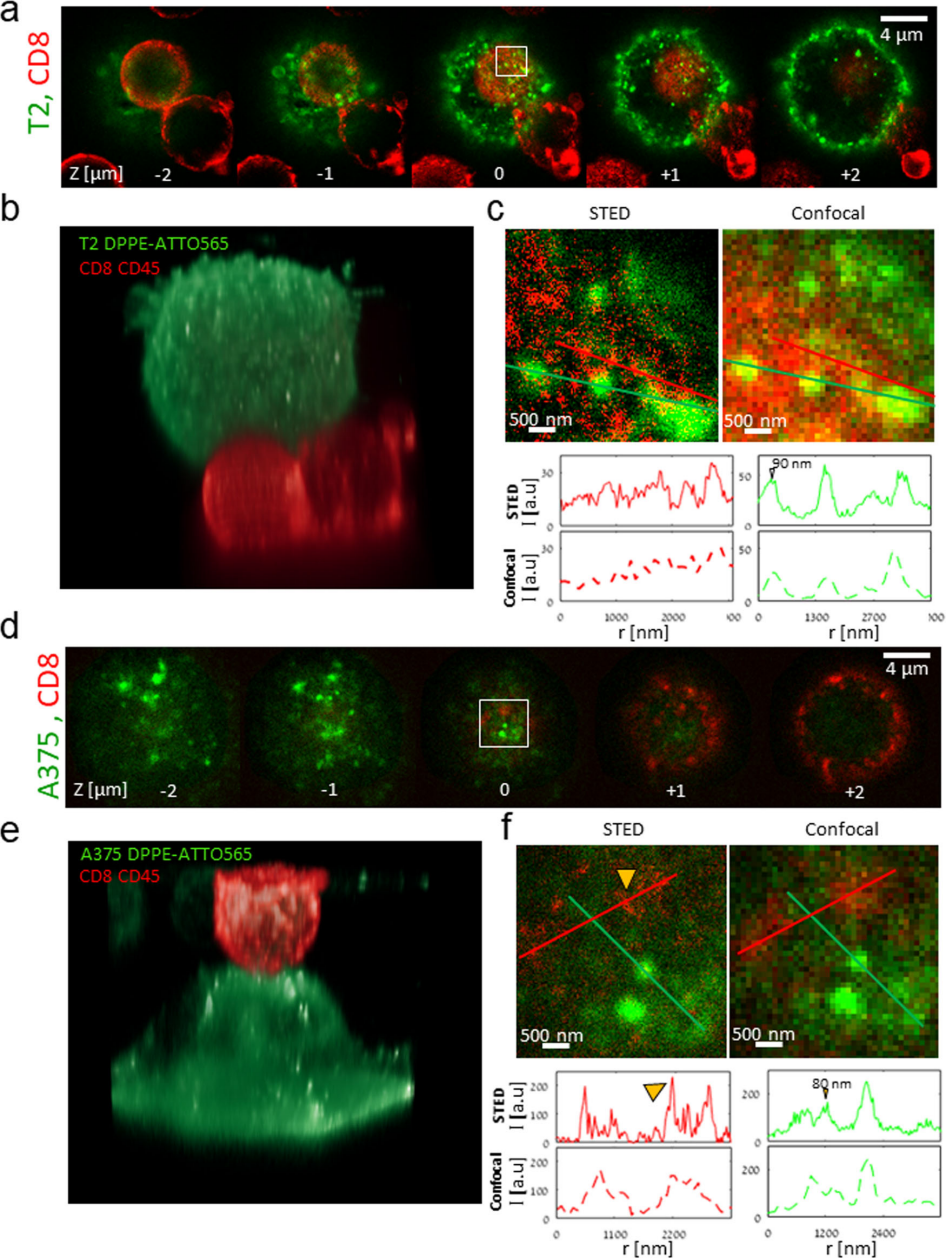
STED microscopy of CD8^+^ T cell conjugates with APCs and melanoma cells. **a** STED images of cell conjugates of CD8^+^ T cells with T2 cells loaded with the activating peptide NY-ESO-1. The PM of the CD8^+^ T cells was stained for αCD45 and Alexa647 (red) and the PM of the T2 cells was stained using DPEE-Atto565 (green). Images are shown at different heights (focal planes) relative to the cell interface. **b** Three-dimensional rendering of the cell conjugates in panel **a**. **c** Zoom images of the confocal and STED images are shown for the zoom area in panel a at z = 0 (middle image). Intensity of either green or red channels is shown across line profiles (indicated as colored lines, respectively) in the top-row images. The width of small objects (peaks) of interest are indicated. **d** STED images of cell conjugates of CD8^+^ T cells with A375 (melanoma) cells. The PM of the CD8^+^ T cells was stained for αCD45 and Alexa647 (red) and the PM of the A375 cells and the A375 cells were stained using DPEE-Atto565 (green). Images are shown at different heights (focal planes) relative to the cell interface. **e** Three-dimensional rendering of the cell conjugates in panel **d**. **f** Zoom images of the confocal and STED images are shown for the zoom area in panel **d** at z = 0 (middle image). Intensity of either green or red channels is shown across line profiles (indicated as colored lines, respectively) in the top-row images. Yellow arrowheads indicate features of interest in the image and on the intensity line profiles. The widths of small objects (peaks) of interest are indicated.

We started by confocal imaging of a fixed interface between CD8^+^ T cells with T2 APCs that were loaded with the NY-ESO-1 peptide for generating an IS (Fig. 2a, b). Specifically, we imaged the abundant membrane protein CD45 on the CD8^+^ T cells and the membrane of the T2 cells via DPEE staining. The two-color images clearly show the two cells and their interface in multiple z-sections in steps of 1 μm (Fig. 2a). A side view of the 3D representation is shown in Fig. 2b.

To demonstrate SRM of these interfaces, we also operated the confocal microscope in STED mode (Fig. 2c). This mode could resolve nanoscale features such as CD45 clusters at the plasma membrane of the CD8^+^ T cell membrane, adjacent to bright ~90 nm membrane patches on the surface of the T2 cells. These patches are cross-sections of cell protrusions that can be clearly seen in panel a. The point-spread-function (PSF) of the STED microscope was tested using 40 nm crimson beads (Fig. S4a, b) and had a width (sigma, in the lateral plane) of 73.5 nm (Fig. S4c, d). This implies a STED resolution of ~60 nm for a point emitter.

To check this interface, we also imaged the interaction between CD8^+^ T cells with melanoma (A375) cells (Fig. 2d). These cells were also fixed prior to imaging and stained similarly to the T2 cells. Here, the interface was less developed in comparison to the IS of the cells with T2 (compare panels a and d). In the melanoma cells, we could distinguish CD45 clusters on the surface of the interacting T cells (arrowheads in panel f). We note that our staining of CD45 was limited to the surface of the cells. Thus, CD45 appearance in 2D cross-sections in planes +1 and +2 in Fig. 2d is likely due to the ruffled T cell membrane (also evident in Fig. 2e).

Thus, our imaging assay can resolve the interface between various cell types using both confocal and STED microscopy, down to a resolution of ~70 nm. Interestingly, we could capture nanoscale CD45 clusters in the CD8^+^ T cells. These clusters were apposed to bright membrane patches (possibly membrane-bound vesicles), at the surface of T2 APCs.

### SMLM imaging of cell conjugates using the opposing coverslips assay

**Fig. 3:**
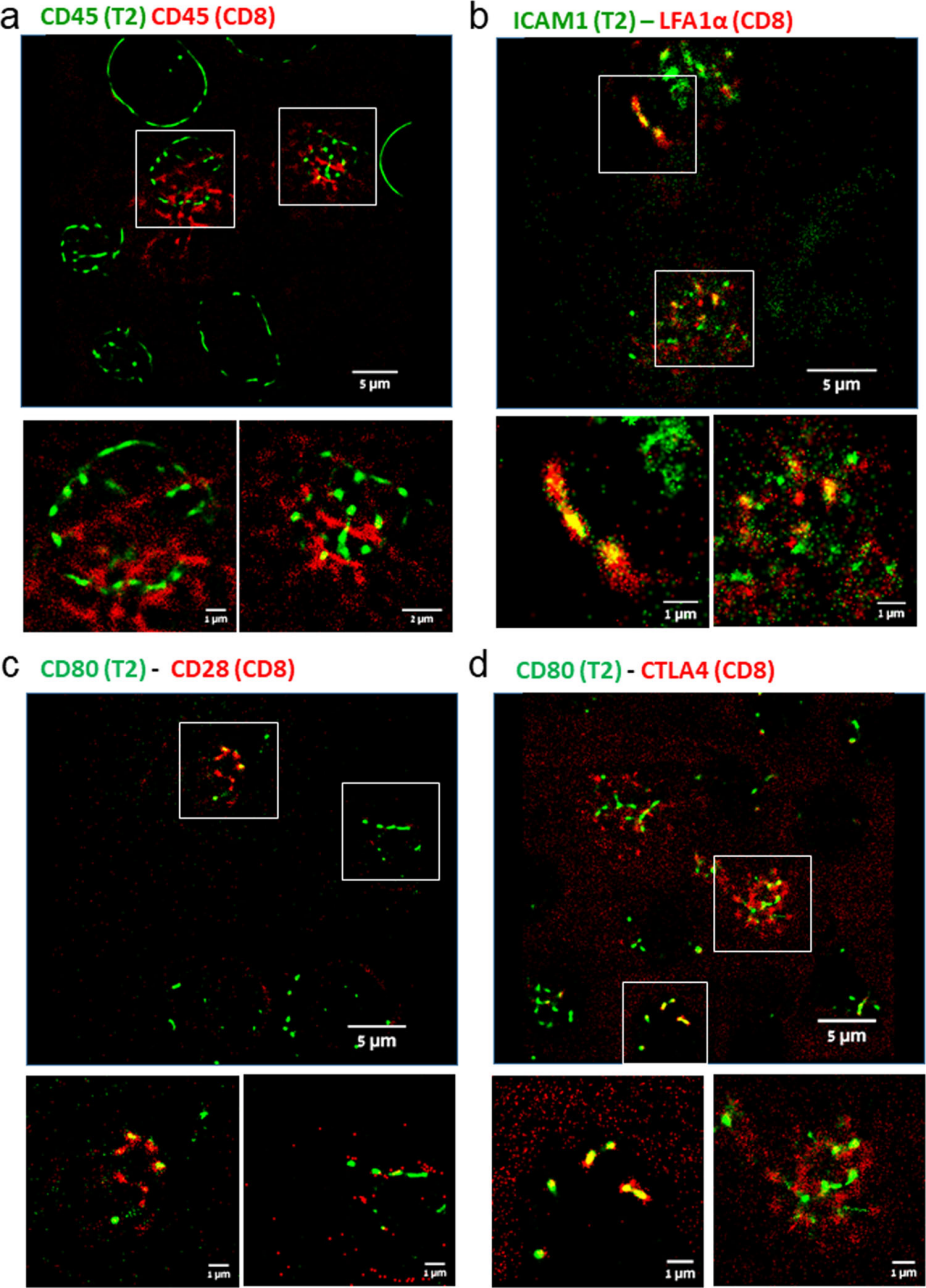
Molecular organization and interactions at the IS. **a**–**d** dSTORM images of cell conjugates of CD8^+^ T cells with T2 cells loaded with the activating peptide NY-ESO-1. **a** The CD8^+^ T (red) and T2 (green) cells were both stained for CD45 (with Alexa647 and Atto488 secondary antibodies, respectively). A representative field (top) and two zoom images of a single conjugate (bottom) are shown. **b** The CD8^+^ T cells (red) were stained for LFA1α, and T2 (green) cells were stained for ICAM1. A representative field (top) and two zoom images of single conjugates (bottom) are shown. **c** The CD8^+^ T cells (red) were stained for CD28, and T2 (green) cells were stained for CD80. A representative field (top) and two zoom images of single conjugates (bottom) are shown. **d** The CD8^+^ T cells (red) were stained for CTLA4, and T2 (green) cells were stained for CD80. A representative field (top) and two zoom images of single conjugates (bottom) are shown.

The confocal configuration allows STED to efficiently reject out-of-focus background. However, such background often compromises other SRM techniques that operate in wide-filed, e.g. SMLM. Importantly, our approach eliminates the need for thick coatings on the opposing coverslips, thus greatly reducing background due to scattering of the excitation lasers and optical aberrations. Thus, we next wanted to demonstrate imaging the IS between live T2 and CD8^+^ T cells via dSTORM. For that we stained the T2 and CD8^+^ T cells with αCD45 and different fluorophores on secondary antibodies (Atto488 for T2 and Alexa647 for CD8^+^ T cells) (Fig. 3a). Distinct membrane ruffles could be detected at the surface of both T2 and CD8^+^ T cells.

We further aimed to visualize specific interactions that are known to occur between surface proteins across the immune synapse between T cells and APCs. For that, we visualized the integrins ICAM1 on T2, and LFA1α on the CD8^+^ T cells (Fig. 3b). These interaction lead to better adherence of the cell conjugate^32,33^. Here, sub-micron domains could be clearly detected at the surface of both cells. Many of these domains, but not all, demonstrated close association across the cell–cell interface. Additional protein-protein interactions occur between CD80 on APCs (here, T2) and both CD28 and CTLA4 on T cells. CD28 is a co-stimulatory molecule that augments TCR activation^34^. In contrast, CTLA4 engagement by CD80 inhibits TCR-dependent activation^35,36^. Here, we observed co-clustering of CD80 and CD28 (Fig. 3c), or with CTLA4 (Fig. 3d) across the IS in some cell conjugates, but not in all (compare zoom regions of individual conjugates in each panel).

Our SMLM imaging resulted in distributions of localisation parameters and uncertainties that were overall similar for cells-on-cells and for cells on functional coverslips that were imaged in total internal reflection fluorescence (TIRF) mode (Fig. S5). For cells-on-cells stained by either Atto488 or DiO, we note a bimodal histogram of localization uncertainties, often with a primary mode at 10–20 nm, and a shoulder (or a secondary mode) at 30–40 nm (see SI for details on imaging and analyses). Notably, the related resolution is comparable to the resolution obtained by TIRF imaging at the interface of the cells with functionalized coverslips (e.g. ref. ^37^). Fourier-Ring Correlation analysis of our dSTORM imaging yielded an effective resolution of <34 nm (Fig. S4e; see details regarding FRC analyses in Methods). Additional bimodality could be observed in the histograms of the Sigma parameter for Atto488 and for DiO. Localizations with Sigma >650 nm were enriched at the center of aggregates and were thus filtered out throughout the study (see Methods).

**Fig. 4:**
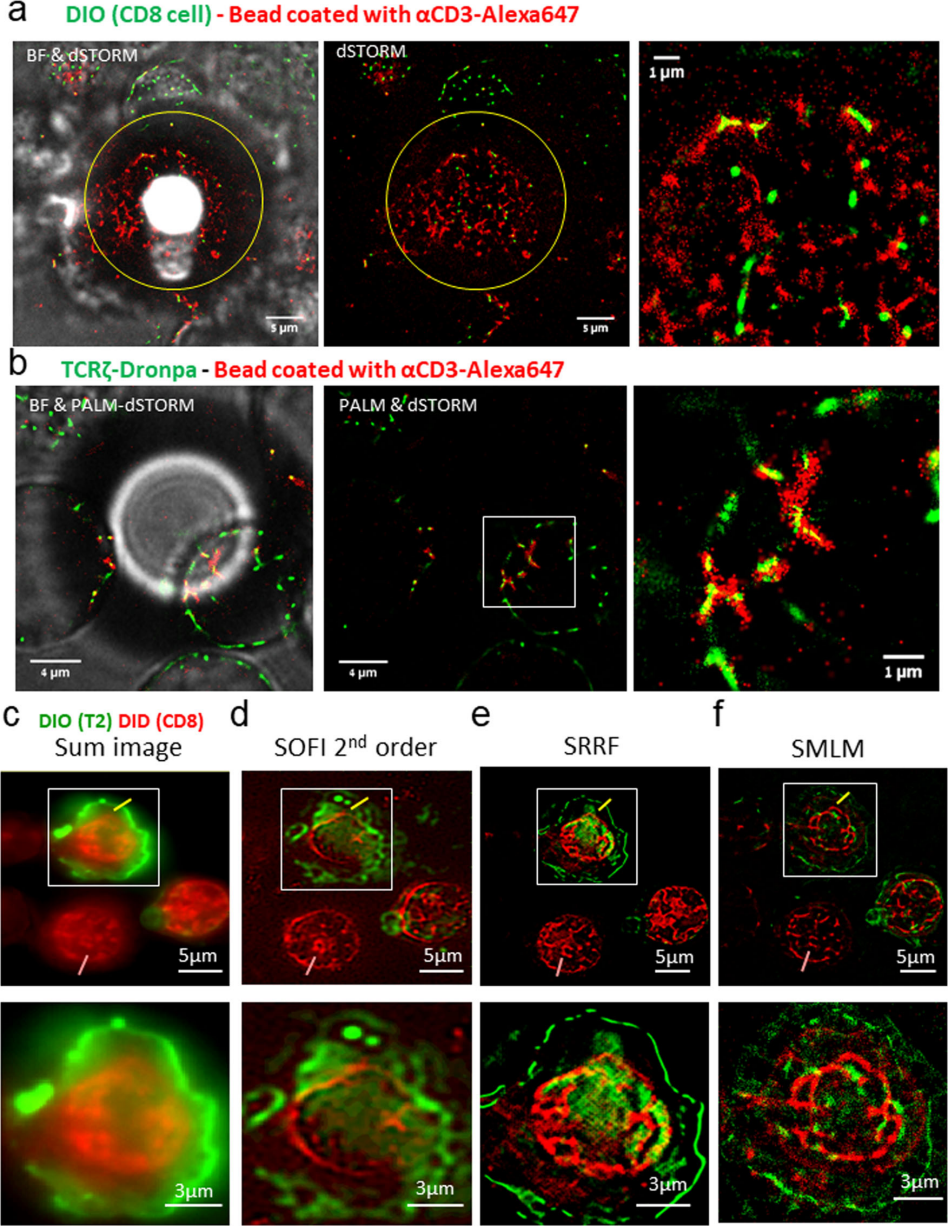
Molecular organization at cell-bead interfaces and using multiple super-resolution reconstruction techniques. **a** The interface between 20 μm silicon beads and CD8^+^ T cells. The CD8^+^ T cells were stained with DIO (membrane dye) and adhered to the bottom coverslip. Twenty-micrometre silicon beads (highlighted diameter in yellow circle) were coated with αCD3ε-Alexa647, and adhered to the top coverslip. A representative cell-bead interface is shown. Bright field of the cell-bead interface, merged with the two fluorescence channels (left panel). Fluorescence imaging of DIO (green) and αCD3ε (red) (middle panel). Zoom in on the cell-bead interface (right panel). **b** The interface between 20 μm silicon beads and Jurkat E6.1 T cells. Jurkat E6.1 (CD4^+^ T) cells stably expressing TCRζ-Dronpa adhered to the bottom of coverslip. On top, 20-μm silicon beads coated with αCD3ε-Alexa647. A representative cell-bead interface is shown. Bright field merged with fluorescence (left panel). Fluorescence of Dronpa (green) and CD3ε (red) (middle panel). Zoom in on the cell-bead interface (right panel). **c**–**f** Super-resolution images of cell conjugates of CD8^+^ T cells with T2 cells loaded with the activating peptide NY-ESO-1. The PM of the CD8^+^ T cells was stained with DiD (red) and the PM of the T2 cells was stained with DiO (green). Reconstructions of the same cells are shown through either **c** Sum image (via summing the intensity of all frames), **d** Second-order SOFI, **e** SRRF, **f** SMLM (via the ThunderStorm software). Top images show the entire field, while bottom images show a zoom on a single-cell conjugate. Yellow and pink lines in top images indicate line cross-sections along which intensity profiles are shown and compared in Fig. S7. See main text and methods for further details regarding the reconstruction techniques.

The cell–cell interfaces formed using our approach are often irregular and may be of interest at a range of focal planes. Thus, we further wanted to test our approach and its imaging performance for more regular interfaces. For that, we conjugated 20 μm spheres adhered to a top coverslip with T cells adhered to the bottom coverslip (Fig. 4a, b). The spheres were labelled with αCD3ε-Alexa647 antibody, and could be clearly observed by both bright-filed (BF) imaging and by dSTORM. In one set of experiments, CD8^+^ T cells were highlighted with the non-specific PM dye DIO (Fig. 4a). Alternatively, we conducted photoactivated localization microscopy (PALM) of CD4^+^ T cells, expressing TCRζ-Dronpa^23^ (Fig. 4b). Interestingly, the TCRζ-Dronpa molecules co-localized with the αCD3ε antibodies, suggesting that they could specifically bind and aggregate them across the surface of the beads. Thus, we show that our imaging approach can also combine dSTORM and PALM for 2-color SMLM.

The imaging details captured by our approach could be compared to the details that can be captured using the traditional approach of imaging cells side-by-side. For that, we first imaged T cells and APCs, both with labelled CD45 with different colors (Fig. S6a). The interface between the cells can be clearly observed as two touching thin lines, but no details of molecular organization can be captured within this interface (Fig. S6 right panel). Likewise, the side-by-side interface of T cells and 20 μm spherical beads also showed relatively lower level of details in comparison to the bead-on-cell images (compare Fig. S6B with Fig. 4a).

Additional approaches have been demonstrated to reconstruct experimental data of fluctuating emitters that is suitable for SMLM. Such approaches include SOFI^38^, its combination with SMLM^39^ and SRRF^40^. Thus, we further tested the performance of these approaches on the same experimental data (Fig. 4c–f), as compared to diffraction-limited imaging (provided by the sum intensity images; Fig. 4c and by SMLM; Fig. 4f). We chose membrane ruffles as our target features (as shown in Fig. 4c). We show that our approach also allows (2nd order) SOFI reconstruction of the same data (Fig. 4d), resulting in a spatial resolution of ~100 nm (e.g. smallest features in Fig. S7d), and improved background rejection over diffraction-limited microscopy^39^ (Figs. 4d, S7a, b). SRRF seems to emphasize feature edges (Fig. 4e). The FRC resolution of this method was estimated at ~160 nm (Fig. S4f). SMLM provided the images with the highest resolution (again ~25 nm) and in single molecule detail. A comparison of the reconstructions along line profiles across distinct features (yellow and pink lines) is provided in Fig. S7c. Background in SMLM reconstruction could be further reduced by synergistically combining SOFI and SMLM reconstruction^39^ (Fig. S7d).

### Imaging early IS formation between live lymphocyte/APC conjugates

**Fig. 5:**
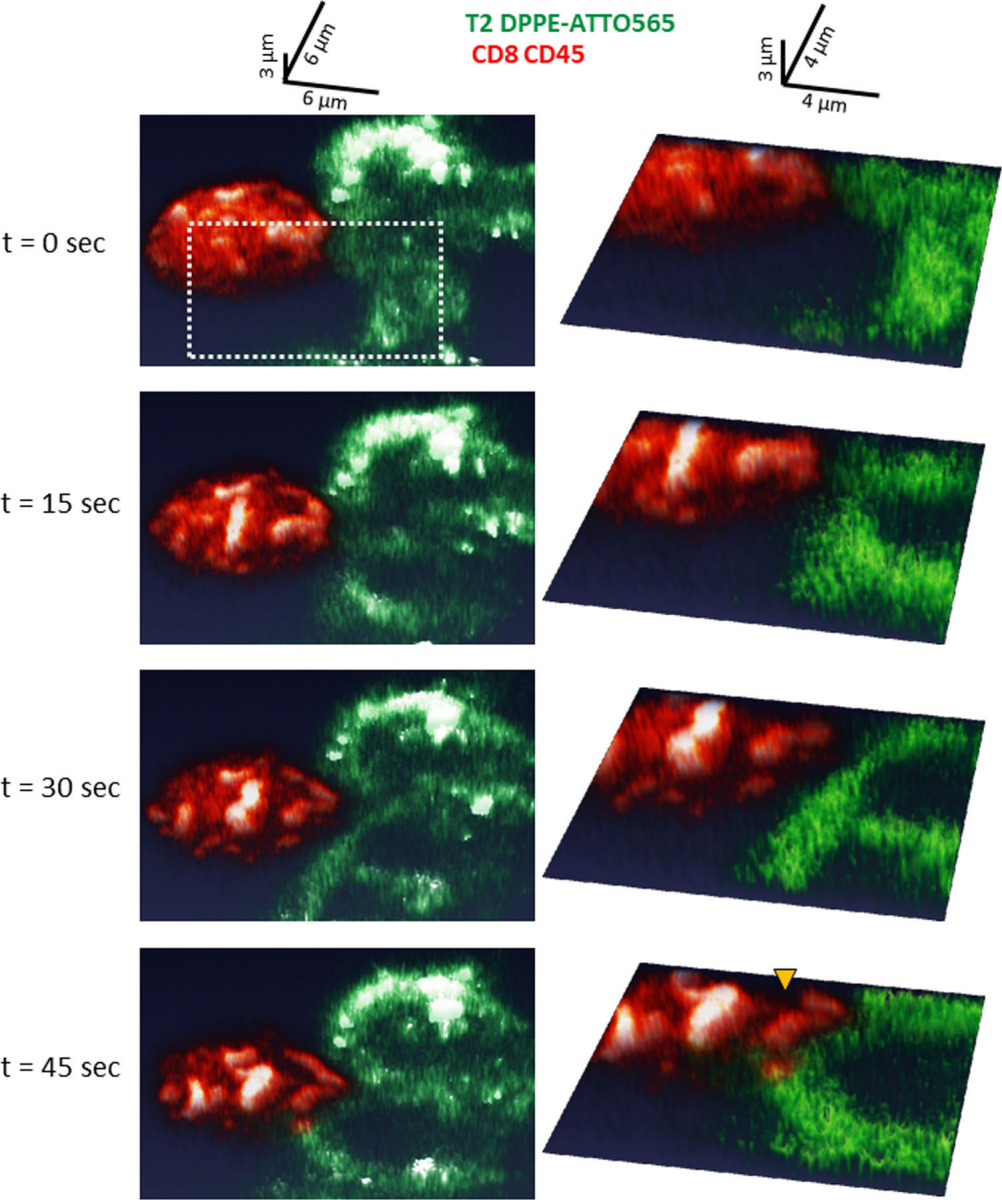
Confocal imaging of a live CD8^+^ T cell interacting with a T2 APCs. Live-cell confocal imaging of conjugates of CD8^+^ T cells with T2 cells loaded with the activating peptide NY-ESO-1. The PM of the CD8^+^ T cells was stained for αCD45 with Alexa647 (red) and the PM of the T2 cells was stained using DPEE-Atto565 (green). Images are shown at different timepoints (t = 0, 15, 30, 45 s) from the start of the interaction (t = 0). Whole field (left) and zoom images (right) are shown and rendered in two different aspects (see axes at top), to better highlight the 3D nature of the interface. All of these 2D images are projections of 3 μm slices. The yellow arrowhead in bottom-right zoom image highlights a lamellar protrusion that facilitates the interaction between the cells.

Using the newly developed conjugation techniques, we set out to image the IS between live T/APC conjugated cells with high-resolution microscopy. Scanning electron microscopy (SEM) images^41^ and recent results from our lab^37^ indicate that the initial contacts between T/APC conjugates occur via lamellae and cell protrusions. We conducted confocal imaging of the early formation of the IS between T2 (stained with DPEE) and CD8^+^ T cells (stained for CD45). Our imaging could capture the first contact between these cells through CD8^+^ T cell protrusions (Fig. 5; yellow arrowhead in zoom image). In this interface the upper red (CD8^+^) cell approaches the bottom green (T2) cell from its upper left side. Thus, an interface is formed with a significant horizontal overlap between the cells. The temporal resolution of this imaging was 5 s per 3D constructed image (15 × 6 × 5 µm). The images were oriented for improved impression of the contact evolution over time.

We further show the interface of live CD8^+^ T cells and T2 cells, as imaged by 2-color dSTORM. Imaging was conducted of cells that form the conjugation either side-by-side (Movie M1; CD8^+^ cells in green and T2 cells in red), or one on top of the other (Movie M2; CD8^+^ cells in red and T2 cells in green; The middle cell conjugate is the one shown in Fig. 4c–f). While only the PM of the cells is visible in Movie M1, a reach nanoscale patterning is visible in movie M2. See further details on movie reconstruction in the Methods.

## Discussion

High and super-resolution imaging of cell conjugates have been hindered by the inappropriate orientation of the interface in typical microscopy. Here, we developed a simple way to form the interface between the cells in an orientation that is parallel to the coverslip and thus, favourable for fluorescence microscopy. We demonstrate our method by visualizing the IS between CD8^+^ T cells and APCs (T2 hybridoma cells, loaded with NY-ESO-1 peptides). To verify the compliance of our approach with the physiological formation of the IS and its function, we measured Ca^++^ influx in T cells, and cell killing upon engagement of the APCs loaded with the cognate peptide. Our SRM imaging could resolve multiple features that were otherwise undetectable (or barely detectable) by diffraction-limited microscopy. Such features included membrane ruffles, nanoscale CD45 clusters and dense membrane domains, closely associating nanoscale integrin domains, and the engagement of APCs by cell protrusions of the CD8^+^ T cell. Importantly, we demonstrate imaging of the interaction of the CD8^+^ T cells with melanoma (A375) cells, showing our approach’s broad applicability to multiple cell–cell conjugates.

We showed that our method is compatible with a wide range of microscope modalities. These include wide-field and confocal arrangements, diffraction-limited and super-resolution microscopy, as well as fixed- and live-cell imaging. Imaging covers spatial ranges from large-scale microscopy for quickly identifying forming cell conjugates, to single-cell conjugates, and even to single molecules at the interface. Super-resolution imaging and reconstruction techniques include STED, SMLM (dSTORM and PALM), SOFI, and SRRF. The spatial resolutions obtained were comparable to imaging of single cells using these techniques. The high resolution of our imaging attest to the lack of optical aberrations and high mechanical stability of our approach. Our imaging typically includes 2 colors, for distinguishing various molecules and entities on the two interacting cells, but can be easily expanded to more colors.

We also tested alternative approaches that held the potential for wide-field super-resolution microscopy, including a cell-trapping approach previously demonstrated using confocal imaging^15^. We found that it is compatible with STED imaging, but not with SMLM or other wide-field imaging techniques due to the high background of the capturing apparatus (wells, or porous membrane). Our approach of imaging cell conjugates using opposing surfaces is a newer and relatively simpler approach that is more compatible with wide-field SRM, including methods such as SMLM, SOFI and SIM (Table 1).

Compared to current cell-trapping approaches (Table 1), our method shows the following advantages: elimination of background and optical aberrations due to thin coatings and very high mechanical stability, which is a particular advantage over the cell traps technique. These properties enable SRM imaging in both confocal (STED) and wide-field (SMLM, SIM, SOFI) configurations. Resolution in all images were comparable to imaging of the interface of the cells with artificial coverslips (e.g. ref. ^37^). While the cells are immobilized on the coverslips, their interacting sides remain free. This eliminates other problems, such as the considerable force applied by optical traps. Furthermore, our approach is simple, low-cost, widely compatible with many cell types, and with various microscopes (without the need for modification). It also shows an increased yield in imageable conjugates in comparison to optical traps. Specialized coatings of the coverslips can serve to optimize the adherence and functional properties of the surfaces, including Poly-L-Lysine, gold beads as fiduciary markers, various antibodies, fibronectin and more.

Recently, the introduction of lattice light sheet microscopy (LLSM)^42^ to the imaging of cell–cell conjugates has allowed very fast imaging of the evolving interface (1.3 sec for 130 z-cross-sections)^43^. The lateral spatial resolution is 230 nm and the z-resolution is 370 nm^42^. Thus, it improves the z-sectioning capability, yet it is still far from the resolution that we show for features within the imaged interfaces. The clear advantage of the LLSM technique over our approach is its very fast time resolution of the entire cell^44^ or of the cell–cell conjugate^43^.

We expect that the pragmatic use and combination of reconstruction techniques can improve and optimize the super-resolution reconstruction of data collected with our assay. This is especially important when using dim fluorophores and for fast live-cell imaging (e.g. ref. ^39^). Moreover, we have recently published a simulation of cell–cell interfaces^45^. Together with the imaging approach presented here, these tools form an effective toolbox for imaging and simulating cell–cell interfaces. For instance, datasets from imaging can be directly plugged into the simulation and serve as constraints or as ground-truth data for hypothesis and prediction testing^37,45^.

In the context of T cells, early diffraction-limited microscopy has shown striking patterns of molecular organization within the synapse^46^. However, difficulties with imaging cell–cell interfaces have led researchers to develop and employ multiple artificial interfaces to mimic the APCs. Indeed, these interfaces have produced much of our current knowledge about the microscale organization of the IS and its dynamics. These model systems, however, do not recapitulate the dynamic 3D architecture of the signalling and endocytic complexes in the IS. For instance, there are several key properties of the IS that suggest that it cannot be fully mimicked by model interfaces. First, APCs may actively contribute to synapse formation and regulate the localization of surface molecules^47^. Second, cytoskeletal control of membrane stiffness, shape and movement can generate forces between surface proteins of the interacting cells. These forces, together with the local membrane environment, have been recently shown to markedly influence the affinity of the interactions between the TCR and the peptide-MHC (pMHC) molecules^48,49^. Third, there are various types of APCs with a number of functional states that differ in their capacity to stimulate lymphocytes. Thus, studying the IS in real cellular interfaces is crucial to our mechanistic understanding of antigen presentation and lymphocyte activation^50^.

Importantly, routine and robust imaging of cell–cell interfaces holds the potential to study and screen for optimal T cells for adoptive immunotransfer for immunotherapy in the clinic. Specifically, signalling by chimeric antigen receptor (CARs) and their optimization, or the search for neo-antigens, would greatly benefit from our simple, yet robust and widely applicable imaging approach at the single-cell and single molecule resolution. Thus, we expect that our techniques will become an important tool in the study of such intercellular interfaces for basic research and for clinical applications.

## Methods

### Cell lines

Jurkat J76 (CD8^+^) and T2 hybridoma cells were a kind gift from the Acuto lab at Oxford. A375 melanoma cells were obtained from the Samules lab. Jurkat E6.1 and Raji B cells were obtained from the Samelson lab (NIH), and OT-1 and AND cells were obtained from the Schwarzberg lab (NIH).

Jurkat E6.1 cells, stably expressing TCR*ζ*–Dronpa or stably expressing PAGFP-actin, were available for this study from previous work^23^.

### Sample preparation

For visualizing conjugated cells in a horizontal orientation, we attached the different types of cells to different glass surfaces that were placed one on top of the other. Twenty-four hours prior to the experiment, T2 hybridoma cells were treated with NY-ESO-1 peptide for MHC-I loading. Two types of glass surfaces were prepared: the 8 wells ibidi #1.5 glass chambers and small glasses that fit the opening of the chamber well. Both of the surfaces were cleaned and treated according to a previously described technique^31^. Briefly, chambers and small glasses were washed with acidic ethanol at room temperature (RT) for 10 min; liquid was then aspirated and coverslips were dried at 37 °C for 1 h. Cleaned coverslips were incubated at RT for 15 min with 0.01% poly-L-lysine (Sigma) diluted in water. Liquid was aspirated and coverslips were dried at 37 °C for 2 h. Coverslips were subsequently incubated with non-stimulatory antibodies at a concentration of 10 μg ml^−1^ overnight at 4 °C or 2 h at 37 °C. Finally, coverslips were washed with phosphate buffered saline (PBS).

### Samples of cell-bead interfaces

Beads, 20 μ in diameter (47148-10, Carposcular) were attached to the upper coverslip by poly-L-lysine coating. Prior imaging, the beads were stained with an αCD3ε-Alexa647 conjugated antibody (FAB100R, R&D Systems).

### DiD, DiO membrane staining

The plasma membrane was tagged by incubation of the cells in staining solution containing 10 μM DiD or DiO (Vybrant® DiD Cell-Labeling Solution, Invitrogen, V22887), in PBS for 0.5–5 min. After staining, cells were washed and suspended in imaging buffer.

### Immunostaining

Antibodies were used following the manufacturers’ protocols. Briefly, 0.5 µg of antibody was added to 500 × 10^3^ cells suspended in FACS buffer (90% PBS 10% FBS 0.02% Na-Azide) for 30 min on ice. Then cells were washed twice in PBS and suspended in 1.5 ml of FACS buffer (90% PBS 10% FBS 0.02% Na-Azide) containing 1 µg of secondary antibody. Cells were washed and suspended in imaging buffer (RPMI without phenol red, 10% FBS, 25 mM HEPES) for live-cell imaging.

The antibodies used in our experiments include:

Mouse anti human CD45 (BD Pharmingen, PMG555480)

Mouse monoclonal IgG1 αCD45-Alexa647 (BioLegend, 304056)

Mouse monoclonal IgG2a αCD11a (LFA1α) (BD Pharmingen, 555378)

Mouse monoclonal Anti-ICAM1 (Abcam, ab2213)

Mouse monoclonal Anti-CD80 (Abcam, ab86473)

Rabbit monoclonal Anti-CTLA4 (Abcam, ab134090)

Rabbit monoclonal Anti-CD28 (Abcam, ab243228)

Goat anti-mouse Atto488 secondary antibody (Sigma-Merck, 62197)

Goat anti-mouse IgG1 (γ1) secondary antibody, Alexa Fluor 647 conjugate (Life Technologies, A21240)

Goat anti-Rabbit secondary antibody, Alexa Fluor 647 conjugate (Life Technologies, A21244)

### Cell fixation

In fixed cell imaging, cells after staining, were fixed by 2.4% Paraformaldehyde (PFA) for 30 min in 37 °C and washed with PBS.

### Single molecule localization microscopy

Two-color dSTORM imaging was conducted both for fixed and live cells.

Fixed cells were suspended in a STORM imaging buffer^51,52^. Live cells were suspended in Imaging buffer (RPMI without phenol red, 10% FBS, 25 mM HEPES.The imaging was performed using a total internal reflection (TIRF) Nikon microscope with a CFI Apo TIRF ×100 oil objective (NA 1.49, WD 0.12 mm). Imaging in TIRF mode served to visualize molecules at the PM of spreading cells in close proximity to the coverslip (up to ~100–200 nm). Fluorophores were activated using a low intensity laser illumination at 405 nm (~0.5% of 30 mW in maximum), and sequentially imaged in a following frame using laser excitation at either 488 nm, or 647 nm (80–100% of 90 mW in maximum for 488 nm or 200 mW for 647 nm). Laser illumination at all wavelengths covered a circular area with a diameter of 80 μm at the sample. dSTORM acquisition sequence typically took ~2.5 min at 13.4 fps of an EMCCD Ixon^+^ camera. The pixel size was equivalent to 160 × 160 nm^2^ at the sample. Excitation and imaging were performed through a quad dichroic (C-NSTROM QUAD 405/488/561/647/FILTER; Nikon). For two-color SMLM imaging, we used immunostaining as specified for each experiment.

### Large-scale imaging

To get a quick overview of the sample for later cell-on-cell acquisition, we used large-scale imaging by acquiring grids of about 100 fields of view, with single-cell resolution. Imaging was performed using a TiE Nikon microscope (already described for SMLM) using epifluorescent illumination. An area of about 500 µm^2^ was automatically scanned with consecutive fields of view of 80 µm^2^ at three different z planes of −8, 0 and 8 µm relative to the focal plane by using a piezo stage control. Moreover, to distinguish between conjugates on the opposing coverslips, cells were stained and imaged in two-color channels in each z-plane. Finally, after reviewing the acquired image, in-depth 3D imaging was performed at the specific x–y coordinates of cell conjugates of interest with high spatial and axial resolution.

### Confocal/STED microscopy

Two-color confocal and STED imaging were performed using an Abberior STED system (Expert line; Abberior Instruments, Göttingen, Germany) mounted on a TiE Nikon microscope operated by the Imspector software (v0.13.11885; Abberior Instruments, Göttingen, Germany). Samples were imaged with a CFI SR HP Apochromat TIRF ×100 NA 1.49 oil immersion objective (Nikon Instruments). Samples were excited with either a 2 mW 561 nm pulsed laser (60 ps) or with a 2 mW 640 nm pulsed laser (60 ps) at 10%. For STED acquisition an additional doughnut-shaped beam depletion laser of 5 mW 770 nm at 18% was applied with a delay time of 1 ns and with width of 20 ns. Reflected light was detected using two APDs with bandpass filter of 570–630 nm and 650–720 nm. The pinhole was set to 1 Airy unit. Samples were scanned across an area of 600 × 600 pixels, 100 nm^2^ each for the confocal setup or an area of about 800 × 800 pixels, 30 nm^2^ each for the STED setup, with pixel dwell time of 15. Each line was scanned ×1 or ×2 times (confocal/STED) and the signal was the product of the intensity accumulation. For 3D imaging a piezo stage was utilized to scan the area with 1 μm axial resolution.

PSF quantitation for STED images was performed on Abberior crimson beads 40 nm.

### Synchronization of cell–cell encounters

Synchronization of cell–cell encounter was primarily achieved by timing when the two opposing surfaces were placed one on top of the other. Further synchronization can be achieved in our assay by sliding the opposing coverslips relative to each other. The spherical beads (diameter 20 μm, Corpuscular, 147148-10) placed between the surfaces ensure that they remain perfectly parallel during their relative translation. We recommend immobilizing the top coverslip and using the translational degree of freedom of the microscope stage (motor or piezo) for sliding the bottom coverslip relative to the top one. Alternatively, the bottom coverslip can remain fixed in position, while a small manipulator can translate the top coverslip (as drawn in Fig. 1a). In an inverted microscope (as used throughout this study), the top coverslip can be approached from above. Top lighting might need to be tilted for that, or a small translational stage can be mounted on the upper arm of the microscope. We note that care should be taken regarding the proper positioning of the lower coverslip (or chamber) on the sample holder, for avoiding variations of height, and thus, relative separation between the coverslips during the translations.

### Calcium assay

For calcium-flux experiments, CD8^+^ T live cells were loaded with Fluo-4AM (Molecular Probes, F10489) at 5 μM for 60 min in the presence of 2.5 mM probenecid. T cells were transferred to imaging medium (RPMI without phenol red, 10% FBS, 25 mM HEPES) and allowed to adhere to the non-stimulating coverslips. Engagement with live T2 cell previously loaded with a cognate peptide, lead to T cell activation. We quantified Fluo-4 responses by determining the average intensity of a region within each cell as a function of time using the ImageJ program (NIH).

### Killing assay

T2 cells were 24 h loaded with peptide NY-ESO-1 or DMSO (as a control). On the day of experiment, T2 cells were also labeled with DiD. Mixing of CD8^+^ T cells and T2 cells started the experiment. At several timepoints, T2 (fluorescent) cells were counted using Trypan blue staining for dead/live differentiation.

### Statistics and reproducibility

All statistical analyses were conducted using a *t*-test (assuming two-tailed distribution and two sample unequal variance), unless otherwise mentioned. The data consist of representative microscopy images, typically of no less than 10 cells imaged per condition. Samples were collected over no less than two independent experiments (and typically over three experiments).

### Data processing

#### SMLM (dSTORM and PALM) reconstruction

Data acquired by SMLM was analysed by the NSTORM module in NIS-Elements (Nikon) or by ThunderSTORM^53^ for the identification of individual peaks and for grouping them into functions that reflect the positions of single molecules^54^. Next, peaks were grouped and assigned to individual molecules for rendering. Peak grouping used a distance threshold and a temporal gap to account for possible molecular blinking^54^. For fixed cells experiments, a temporal gap of ~50 msec and a distance threshold of 20 nm were applied for each fluorophore separately in order to minimize possible over-counting of molecules. Drift compensation and channel registration were performed using dedicated algorithms in the ThunderSTORM software. For live cells experiments no drift compensation was applied and a distance threshold of 20 nm was taken (regarding time gaps, each image in a live experiment accumulates 2–2.5 sec of acquisition time as will be describe next). Calibration was conducted using 100 nm Tetraspec fluorescent beads (Invitrogen). Three-dimensional decoding was performed using Nikon NSTORM software. Individual molecules are presented in dSTORM images with intensities that correspond to the probability density values of their fitted Gaussian with respect to the maximal probability density values detected in the field.

To generate a frame in a live-cell imaging sequence, accumulation of 50 frames of an SMLM movie with a frame rate of 50 fps was acquired, with alternating acquisition of the green and red channels. Thus, each image represents 1 sec of SMLM acquisition time. The images were assigned the frame time of the first participating frame from the SMLM movie. These accumulated frames were further used to generate movies of the cell conjugates.

For Atto488 and DiO, we detected localizations with relatively large sigma values (>650) that were enriched at the centres of aggregates and were likely misidentified as single molecules. These localizations were filtered out of the presented data.

We provide example movies of cells captured via our cell-on-cell approach. For clarity, the movies are shown separately for our used fluorophores in this study, namely - Alexa647, Atto488, DiO and Dronpa (Movies M3-M6, respectively).

#### SRRF and SOFI reconstruction

We acquired the SRRF images using a published software^40^ with the following parameters: Ring = 0.10, radial magnification = 10, frames per point = 5. Second-order SOFI reconstruction was performed using the Matlab SOFI version provided by^55^ with the default parameters.

#### FRC analyses

The resolution estimate for SMLM reconstruction and SRRF are done using the FRC criterion^56^. Two criteria are being employed: half-bit-threshold^57^ and 1/7 threshold as suggested in the original paper^56^. For SRRF, we compared the result to the SMLM image, as SRRF yields an image and not localization-based data. The resolution estimate for SMLM was 34 nm and the Relative Resolution (for the comparison) is 164 nm. Therefore the estimation for SRRF is: ${\mathrm{Final}}\,{\mathrm{resolution}} = \sqrt {180\,{\mathrm{nm}}^2 - 34\,{\mathrm{nm}}^2} \approx 160\,{\mathrm{nm}}.$

### Reporting summary

Further information on research design is available in the Nature Research Reporting Summary linked to this article.

## Supplementary information

Peer Review File

Supplementary Information

Description of Additional Supplementary Files

Supplementary Movie 1

Supplementary Movie 2

Supplementary Movie 3

Supplementary Movie 4

Supplementary Movie 5

Supplementary Movie 6

Reporting Summary

## Data Availability

The authors declare that the data supporting the findings of this study are available within the article and its supplementary information files, or are available upon reasonable requests to the authors.

## Code availability

No custom code was used. All software and code are available through the cited references.
