# Supplementary material for: Adhering interacting cells to two opposing coverslips allows super-resolution imaging of cell-cell interfaces
Source: Commun Biol. 2021 Apr 1;4:439. doi: 10.1038/s42003-021-01960-2 (PMC8016881; doi:10.1038/s42003-021-01960-2)
Supplement: Supplementary file 2 — Supplementary Information [file 42003_2021_1960_MOESM2_ESM.pdf]

Supplemental Information for:

Adhering interacting cells to two opposing coverslips allows super-resolution imaging of cell-cell interfaces

Julia Sajman[1]*, Yair Razvag[1]*, Shachar Schidorsky[1], Seon Kinrot[1,2], Kobi Hermon[1], Oren Yakovian[1], Eilon Sherman[1]**

[1]Racah Institute of Physics, The Hebrew University, Jerusalem, Israel, 91904
[2]Current address: Graduate Program in Biophysics, Howard Hughes Medical Institute, Department of Chemistry and Chemical Biology, Department of Physics, Harvard University, Cambridge, MA 02138, USA

* - Equal contribution

** - corresponding author

email: eilon.sherman@mail.huji.ac.il

Supplemental Figures and Legends

Figure S1

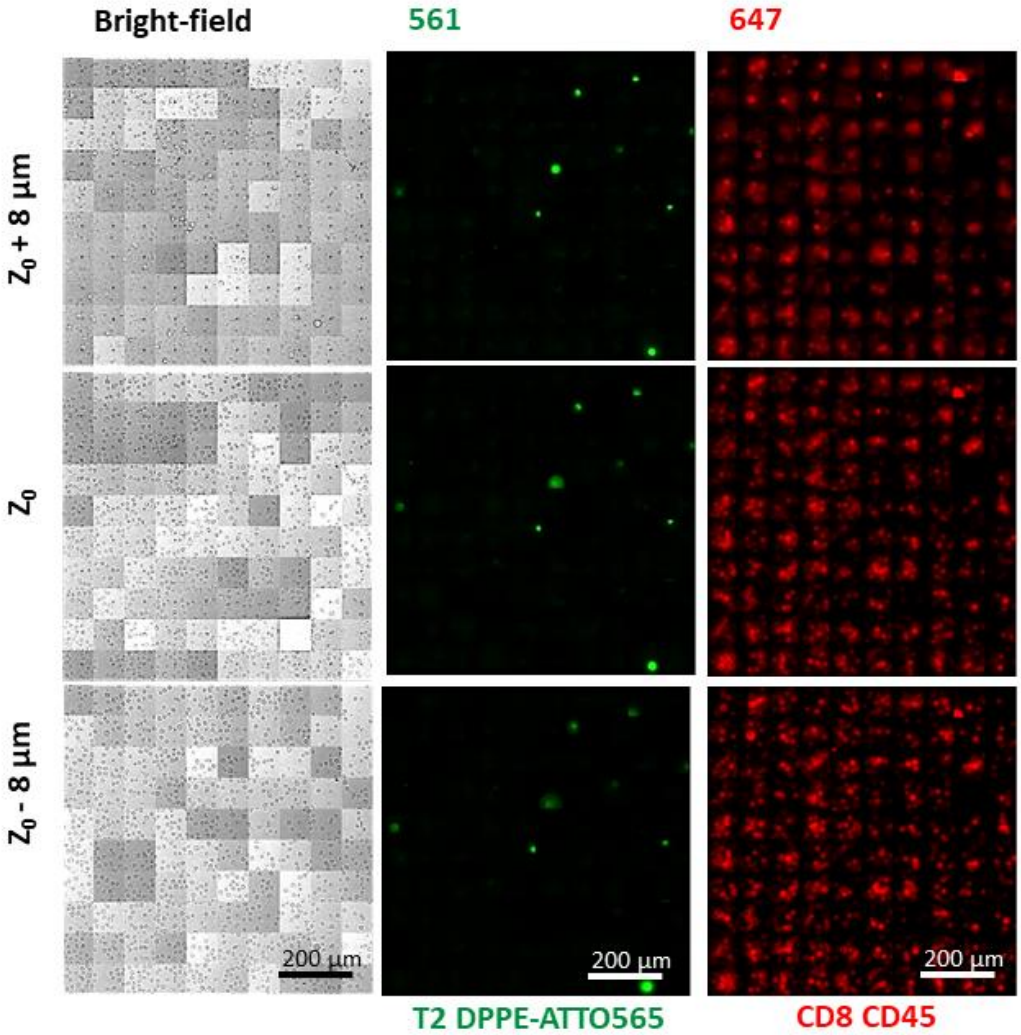

Fig. S1. Large scale microscopy of T/APC cell conjugates on opposing surfaces in multiple height sections

Large scale microscopy images of $CD8^+$ cells with T2 cells loaded with the activating peptide NY-ESO-1. The PM of the $CD8^+$ cells was stained for αCD45 and Alexa647 (red) and the PM the T2 cells was stained using DPEE-Atto565 (green). - Large scale images (a montage of 100 fields) are shown in bright-field (left column). The contrast of these images was adapted here for improved visibility of single cells; (middle and right columns) show each of the two fluorescence channels. Rows are shown for 3 height sections, relative to the expected height of the interface between the cell conjugates (Z_0). The shown images match the data in Fig. 1.

Figure S2

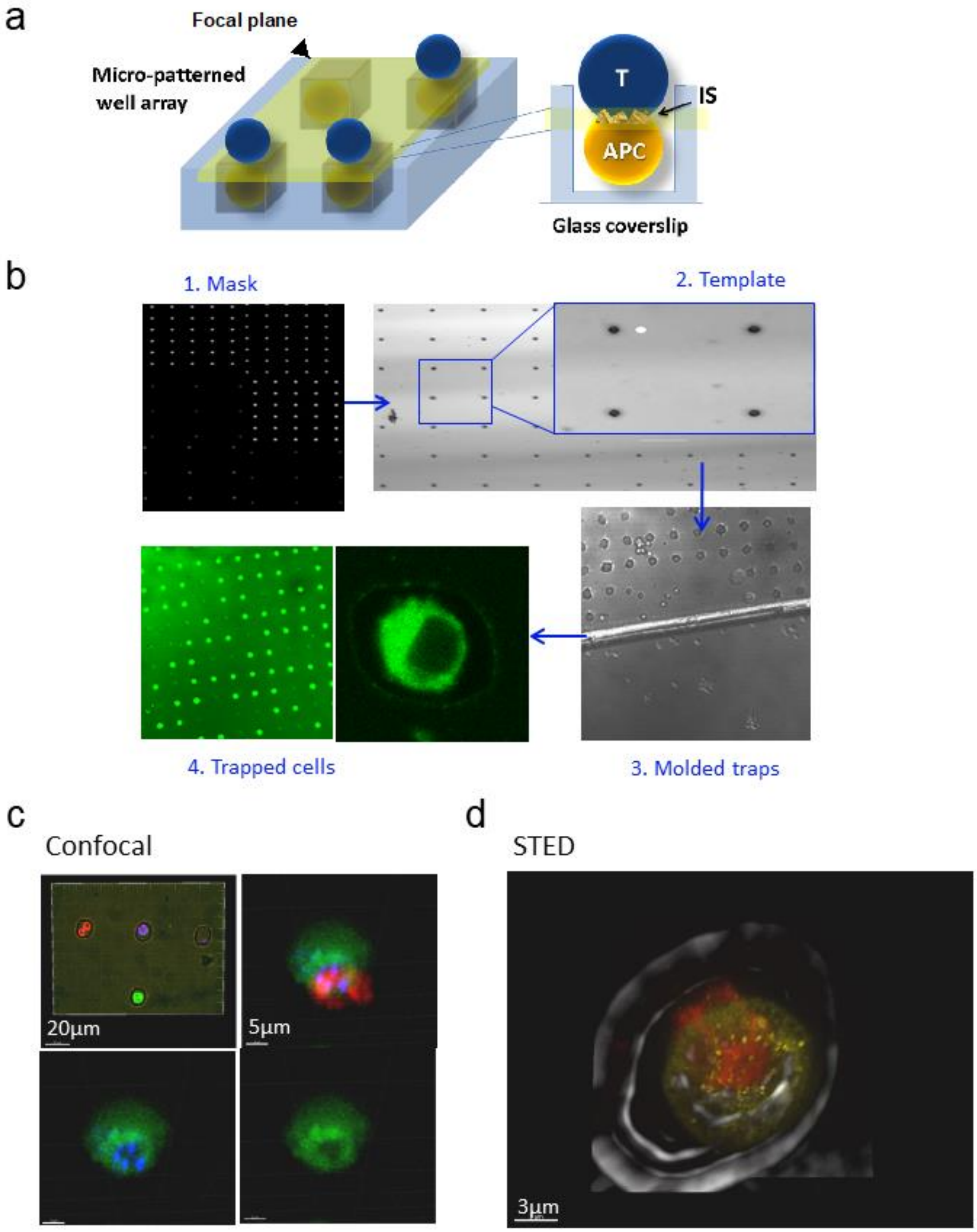

Fig. S2. Trapping and imaging T/APC conjugates in a micro-patterned well array

(a) Schematic description of the cell trapping approach in well arrays molded in a polymer. A micro-patterned well array molded in a polymer of choice is placed on a glass coverslip. The wells trap single APCs and lymphocytes. The immune synapse between the cells is thus aligned in a favorable orientation at the focal plane of a light microscope. (b) Realization of the micro-patterned well traps. Steps (1) Patterning of a mask. (2) Creation of a template using the mask and photolithography. (3) Molding of wells in a curable polymer. Here, in Polydimethylsiloxane (PDMS), The well arrays are molded on Si wafers first and then transferred onto the coverslips. (4) Trapping individual cells of choice in the micro-patterned cell traps. (c) Preliminary confocal microscopy images of single T-cells (AND mouse) conjugated to APCs (B-cells loaded with PCC peptide) in 15μm well traps. T cells expressed GFP-actin (green), B-cells were marked with a non-specific cell marker (red) and both cells were stained with anti-phosphotyrosine antibodies (blue). Scale bars - 5μm, while the top-left panel is 20 μm. (d) STED imaging of T/APC conjugates in micro-patterned traps. The interface between a T cell (Jurkat E6.1 T cell; yellow) and an APC (SEE pulsed Raji B-cell; red) was imaged using emission depletion (STED) microscopy in cell traps (white). A top view with maximal intensity projection of a 3D z-stack is presented. SLP-76 clusters (yellow dots; stained with Atto647 against pY128) could be detected at the interface with resolution down to ~70nm. Scale bar - 3μm. Imaging was performed using a Leica TCS STED microscope.

Figure S3

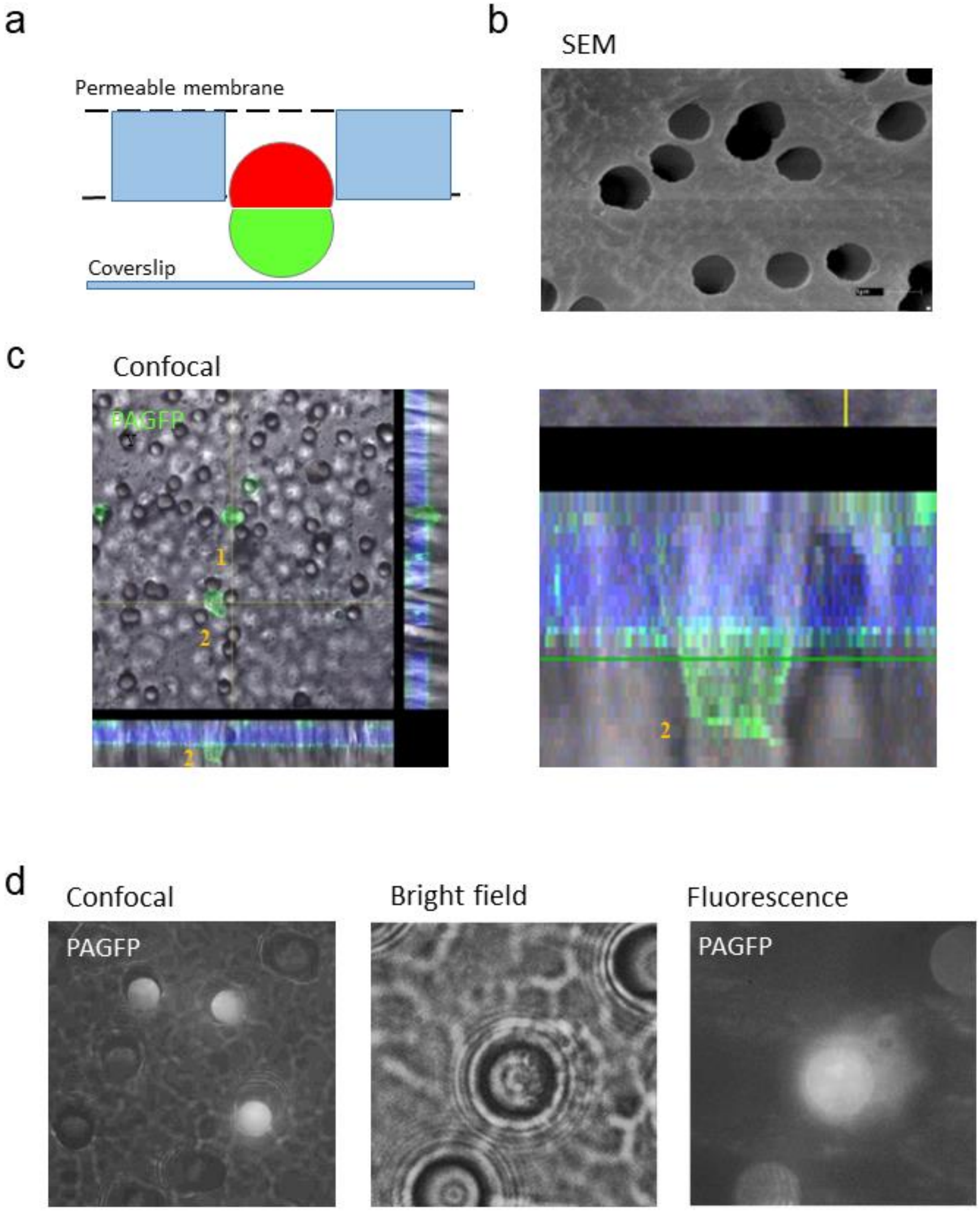

Fig. S3. Trapping and imaging T/APC conjugates in a porous membrane

(a) A schematic description of Trapping and imaging T/APC conjugates in a porous membrane. Cells of one type (e.g. T cells) are first trapped in a porous membrane with 8μm pores (Stelitech Corp, PCT803100,). The membranes are then placed on coverslips, with the adherent conjugate cells (e.g. APCs). The immune synapse between the cells is aligned in a favourable orientation at the focal plane of a light microscope. (b) An SEM image of the porous membrane (top view). (c) Confocal imaging of trapped Jurkat cells in the porous membrane (shown are top and side views in the left and right images, respectively). Cells expressed GFP-actin molecules (in green). Two trapped cells are highlighted and a zoom image of cell #2 is shown (on right). (d) (left) Confocal, (middle) bright field and (right) fluorescence (both wide-field) imaging of T cells, stably expressing PAGFP-actin, and captured in a porous membrane.

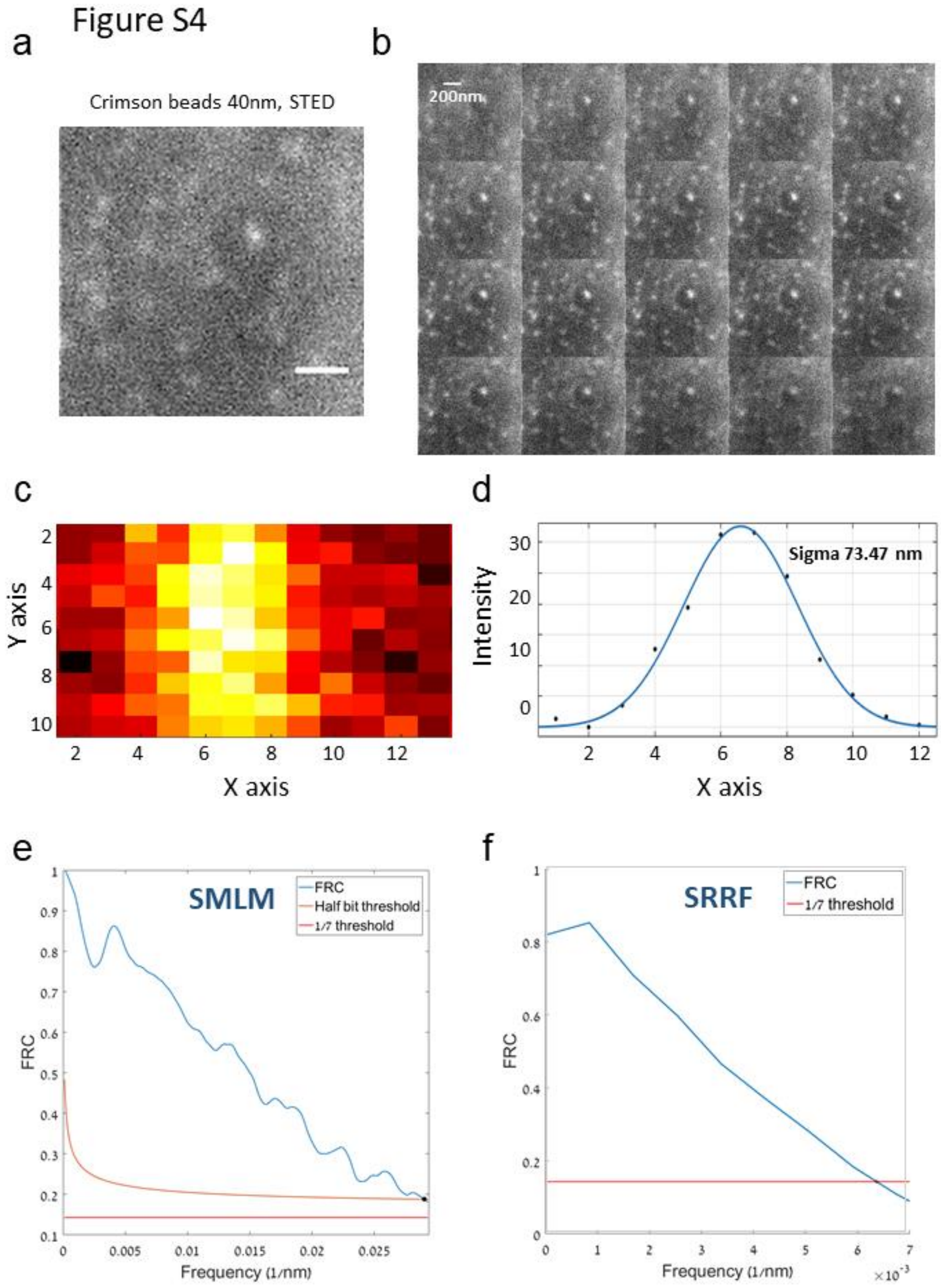

Fig. S4. Resolution of STED, SMLM and SRRF

(a) Crimson beads (40 nm size) imaged by STED in the XY plane. (b) Z stacks of the same image in panel a. (c) Projection of intensity of a single Crimson bead along the x-z plane. (d) Intensity distribution of a single Crimson bead along the Z-axis, showing the width of the convolution between the STED PSF and the bead.

(e) Fourier ring correlation (FRC) of the SMLM reconstruction in Fig. 4f. Blue line: FRC of the experimental data, Orange line: Half-bit threshold estimate (with 34nm cut-off point); Red line: 1/7 threshold. (f) FRC of the SRRF reconstruction presented in Fig. 4e, as compared with the SMLM image, yielding a SRRF resolution estimate of 160nm.

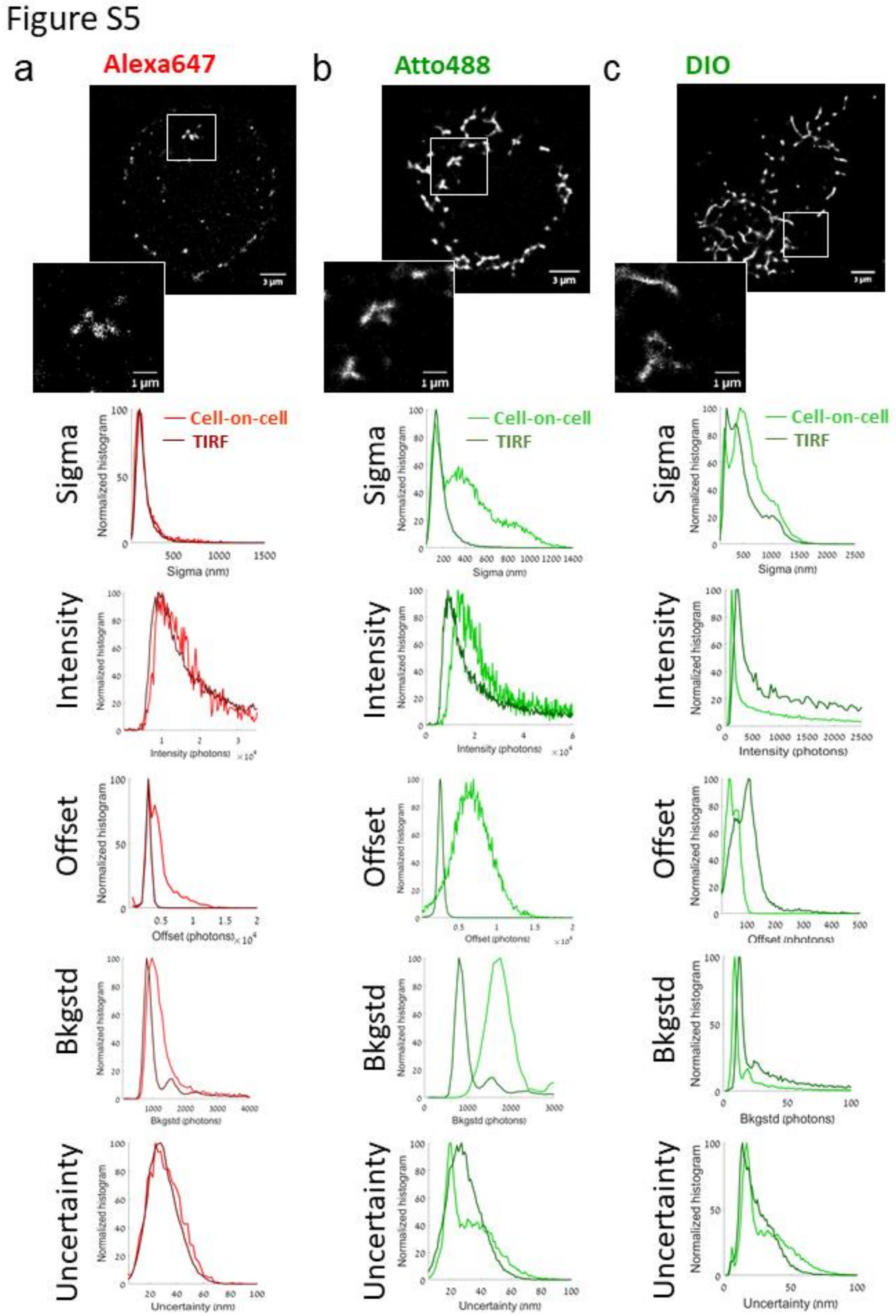

Fig. S5. Localization accuracy of SMLM imaging using opposing coverslips

Data of ThunderSTORM analysis of dSTORM imaging of $CD8^+$ and T2 cell conjugates. Cells were labelled with either (a) Alexa647, (b) Atto488, or (c) DIO. The images were taken from cell conjugates that were imaged using opposing coverslips. dSTORM images of the cellular interfaces are shown. For each fluorophore, histograms are shown of different parameters (light-colored lines), and compared to the histograms obtained for the same colors in TIRF imaging (dark-colored lines). The shown parameters include: Sigma [nm]: standard deviation of the Gaussian fitted to the peak. Intensity [photons]: integrated photons number under the peak (value used to calculate the uncertainty in the Webb/Mortensen formulas). Offset [photons]: baseline of the peak (background absolute value). Bkgstd [photon]: standard deviation of the background (used to calculate the uncertainty in the Webb/Mortensen formulas). Uncertainty_[nm]: standard deviation of the lateral localization uncertainty (used to draw the width of each localization when using Gaussian rendering). The number of localizations for each image was: (a) 4695, (b) 16073, (c) 19866.

Figure S6

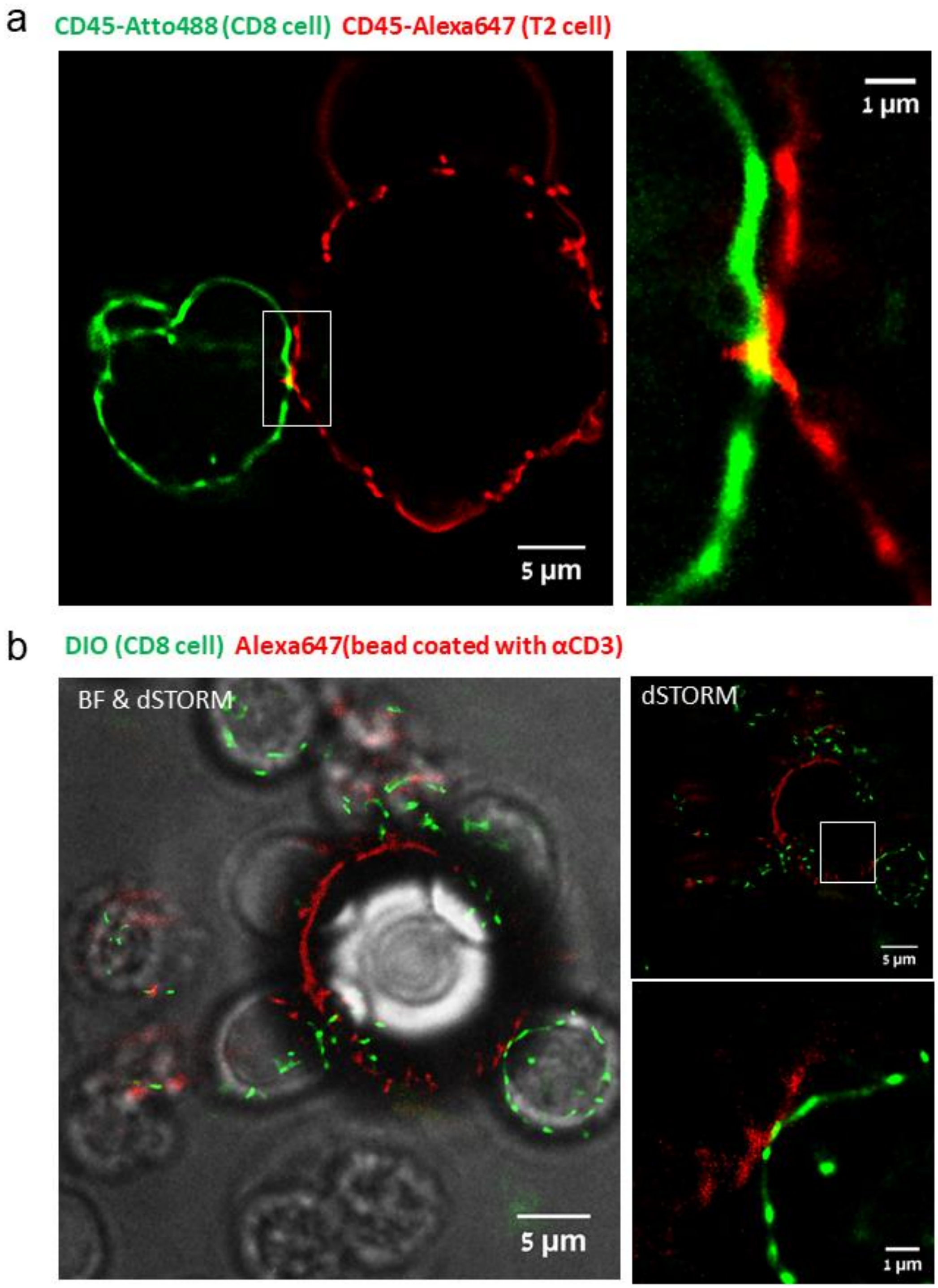

Fig S6. Imaging of side-by-side cellular interfaces

(a) dSTORM imaging of a synapse formed between cell-cell conjugates as they form side by side. The $CD8^+$ T cells were stained with CD45 and Atto488 (green) and T2 cells were stained with CD45-Alexa647 (red). On right, a zoom in on the synapse between two cells. (b) Bright-field (BF) and dSTORM imaging (merged on left) of an interface of a cell and a 20μm silicon bead, as they adhere side by side. $CD8^+$ T cells were stained with DIO (green) and silicon beads were stained with αCD3ε-Alexa647 (red). On right, zoom in on the interface between the bead and the cells.

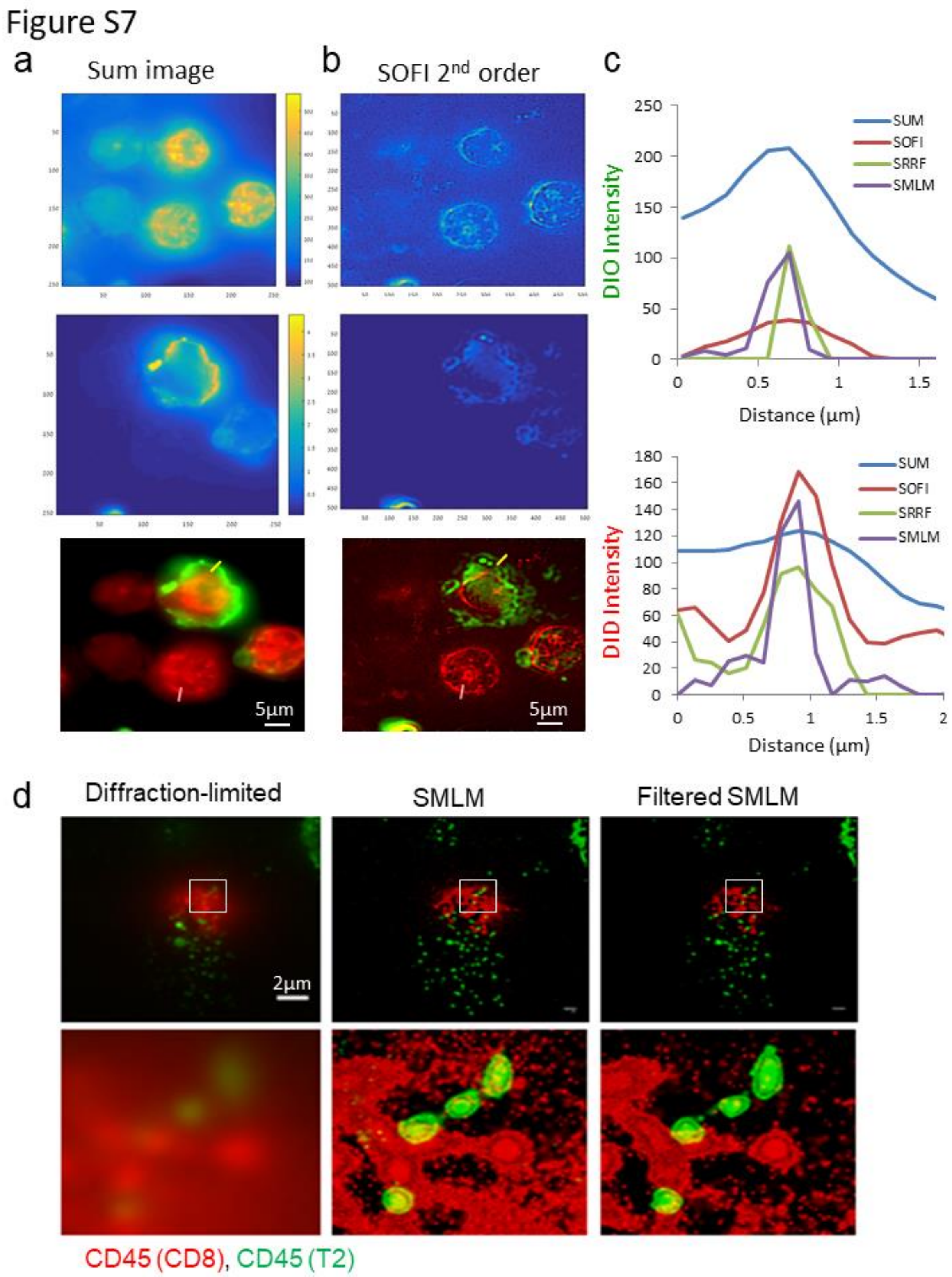

Fig. S7. SOFI-assisted reconstruction

(a) Sum images, and (b) SOFI-reconstructed images of the cell conjugates shown in Fig. 4c and d, respectively. (c) Intensity line profiles across distinct features in the images of Fig. 4d-f for Sum intensity rendering, SOFI, SRRF and SMLM reconstructions. The line profiles are along the yellow and pink lines (for the DiO and DiD stains, respectively) in Fig. 4d-f and in the bottom images of panels a and b (for convenience). (d) SOFI-assisted SMLM reconstruction, of $CD8^+$ and T2 cells loaded with the activating peptide NY-ESO-1. $CD8^+$ cells were stained for CD45 with Alexa488 ($CD8^+$, green) and the T2s cell were stained for CD45 with Alexa647 (red). (bottom row) zoom images. Columns (left to right) are diffraction limited, SMLM and filtered SMLM images.
