# Supplementary material for: Adhering interacting cells to two opposing coverslips allows super-resolution imaging of cell-cell interfaces
Source: Commun Biol. 2021 Apr 1;4:439. doi: 10.1038/s42003-021-01960-2 (PMC8016881; doi:10.1038/s42003-021-01960-2)
Supplement: Supplementary file 3 — Description of Additional Supplementary Files [file 42003_2021_1960_MOESM3_ESM.pdf]

Description of Additional Supplementary Files

File Name: Movie M1. dSTORM imaging of live cell-cell conjugates that form side-by-side. Description: dSTORM imaging of a synapse formed between cell-cell conjugates as they form side by side. The $CD8^+$ T cells were stained with CD45 and Atto488 (green) and T2 cells were stained with CD45 and Alexa647 (red). See methods for details on rendering of the movie.

File Name: Movie M2. dSTORM imaging of live cell-cell conjugates that form one on top of the other. Description: dSTORM imaging of synapses formed between cell-cell conjugates as they one on top of the other. The $CD8^+$ T cells were stained with DID (red) and T2 cells were stained with DIO (green). See methods for details on rendering of the movie.

File Name: Movie M3. dSTORM imaging of live cells as part of conjugates and labelled with Alexa647. Description: dSTORM imaging of a cell cross-section when imaged as a part of a cell conjugate. The cell was stained with Alexa647. The movie was analysed using ThunderSTORM for highlighting localizations (red crosses; see Methods for details on analyses and rendering of the movie).

File Name: Movie M4. dSTORM imaging of live cells as part of conjugates and labelled with Atto488. Description: dSTORM imaging of a cell cross-section when imaged as a part of a cell conjugate. The cell was stained with Atto488. The movie was analysed using ThunderSTORM for highlighting localizations (red crosses; see Methods for details on analyses and rendering of the movie).

File Name: Movie M5. dSTORM imaging of live cells as part of conjugates and labelled with DiO. Description: dSTORM imaging of a cell cross-section when imaged as a part of a cell conjugate. The cell was stained with DiO. The movie was analysed using ThunderSTORM for highlighting localizations (red crosses; see Methods for details on analyses and rendering of the movie).

File Name: Movie M6. PALM imaging of live cells as part of conjugates and expressing TCR -Dronpa. Description: PALM imaging of a cell cross-section when imaged as a part of a cell conjugate. The cell was expressed TCR -Dronpa. The movie was analysed using ThunderSTORM for highlighting localizations (red crosses; see Methods for details on analyses and rendering of the movie).
