# Supplementary material for: Adhering interacting cells to two opposing coverslips allows super-resolution imaging of cell-cell interfaces
Source: Commun Biol. 2021 Apr 1;4:439. doi: 10.1038/s42003-021-01960-2 (PMC8016881; doi:10.1038/s42003-021-01960-2)
Supplement: Supplementary file 10 — Reporting Summary [file 42003_2021_1960_MOESM10_ESM.pdf]

nature research

Corresponding author(s): Eilon Sherman

Last updated by author(s): 21/2/21

Reporting Summary

Nature Research wishes to improve the reproducibility of the work that we publish. This form provides structure for consistency and transparency in reporting. For further information on Nature Research policies, see our Editorial Policies and the Editorial Policy Checklist.

Statistics

For all statistical analyses, confirm that the following items are present in the figure legend, table legend, main text, or Methods section.

n/a	Confirmed	
x		The exact sample size (*n*) for each experimental group/condition, given as a discrete number and unit of measurement
	x	A statement on whether measurements were taken from distinct samples or whether the same sample was measured repeatedly
	x	The statistical test(s) used AND whether they are one- or two-sided<br>*Only common tests should be described solely by name; describe more complex techniques in the Methods section.*
x		A description of all covariates tested
x		A description of any assumptions or corrections, such as tests of normality and adjustment for multiple comparisons
	x	A full description of the statistical parameters including central tendency (e.g. means) or other basic estimates (e.g. regression coefficient) AND variation (e.g. standard deviation) or associated estimates of uncertainty (e.g. confidence intervals)
x		For null hypothesis testing, the test statistic (e.g. *F*, *t*, *r*) with confidence intervals, effect sizes, degrees of freedom and *P* value noted<br>*Give P values as exact values whenever suitable.*
x		For Bayesian analysis, information on the choice of priors and Markov chain Monte Carlo settings
x		For hierarchical and complex designs, identification of the appropriate level for tests and full reporting of outcomes
x		Estimates of effect sizes (e.g. Cohen's *d*, Pearson's *r*), indicating how they were calculated

Our web collection on statistics for biologists contains articles on many of the points above.

Software and code

Policy information about availability of computer code

Data collection	Imspector software (v0.13.11885; Abberior Instruments, Göttingen, Germany), N-STORM module in NIS-Elements (Nikon)
Data analysis	ThunderSTORM (Ref 54), SRRF software (Ref 40) , SOFI - Matlab version (Ref 56), ImageJ, Matlab

For manuscripts utilizing custom algorithms or software that are central to the research but not yet described in published literature, software must be made available to editors and reviewers. We strongly encourage code deposition in a community repository (e.g. GitHub). See the Nature Research guidelines for submitting code & software for further information.

Data

Policy information about availability of data

All manuscripts must include a data availability statement. This statement should provide the following information, where applicable:

- Accession codes, unique identifiers, or web links for publicly available datasets
- A list of figures that have associated raw data
- A description of any restrictions on data availability

Data availability - The authors declare that the data supporting the findings of this study are available within the article and its supplementary information files, or are available upon reasonable requests to the authors.

Field-specific reporting

Life sciences study design

All studies must disclose on these points even when the disclosure is negative.

Sample size	The data consist of representative microscopy images, typically for no less than 10 cells imaged per condition
Data exclusions	No data were excluded
Replication	Samples were collected over no less than two independent experiments (and typically over 3experiments)
Randomization	No grouping was attempted
Blinding	No blinding was attempted

Reporting for specific materials, systems and methods

We require information from authors about some types of materials, experimental systems and methods used in many studies. Here, indicate whether each material, system or method listed is relevant to your study. If you are not sure if a list item applies to your research, read the appropriate section before selecting a response.

Materials & experimental systems

n/a	Involved in the study	
☐	☒	Antibodies
☐	☒	Eukaryotic cell lines
☒	☐	Palaeontology and archaeology
☒	☐	Animals and other organisms
☒	☐	Human research participants
☒	☐	Clinical data
☒	☐	Dual use research of concern

Methods

n/a	Involved in the study	
☒	☐	ChIP-seq
☒	☐	Flow cytometry
☒	☐	MRI-based neuroimaging

Antibodies

Antibodies used

Mouse anti human CD45 (BD Pharmingen, PMG555480)
Mouse monoclonal IgG1 αCD45-Alexa647 (BioLegend, 304056)
Mouse monoclonal IgG2a αCD11a (LFA1α) (BD Pharmingen, 555378)
Mouse monoclonal Anti-ICAM1 (Abcam, ab2213)
Mouse monoclonal Anti-CD80 (Abcam, ab86473)
Rabbit monoclonal Anti-CTLA4 (Abcam, ab134090)
Rabbit monoclonal Anti-CD28 (Abcam, ab243228) Goat anti-mouse Atto488 secondary antibody (Sigma-Merck, 62197)
Goat anti-mouse IgG1 (γ1) secondary antibody, Alexa Fluor 647 conjugate (Life Technologies, A21240)
Goat anti-Rabbit secondary antibody, Alexa Fluor 647 conjugate (Life Technologies, A21244)

Validation

All antibodies were validated by the vendors

Eukaryotic cell lines

Policy information about cell lines

Cell line source(s)	Jurkat J76 (CD8+) and T2 hybridoma cells were a kind gift from the Acuto lab at Oxford. A375 melanoma cells were obtained from the Samules lab. Jurkat E6.1 and Raji B cells were obtained from the Samelson lab (NIH), and OT-1 and AND cells were obtained from the Schwarzberg lab (NIH). Jurkat E6.1 cells, stably expressing TCRζ–Dronpa or PAGFP-actin, were available for this study from previous work (Ref 23).
Authentication	The cell lines were authenticated by the donating labs, as specified above.
Mycoplasma contamination	Cell lines were routinely inspected visually for mycoplasma contamination, but were not tested otherwise for such contamination.
Commonly misidentified lines (See ICLAC register)	N.A.
