# Supplementary material for: Adhering interacting cells to two opposing coverslips allows super-resolution imaging of cell-cell interfaces
Source: Commun Biol. 2021 Apr 1;4:439. doi: 10.1038/s42003-021-01960-2 (PMC8016881; doi:10.1038/s42003-021-01960-2)
Supplement: Supplementary file 1 — Peer Review File [file 42003_2021_1960_MOESM1_ESM.pdf]

Reviewers' comments:

Reviewer #1 (Remarks to the Author):

In this manuscript, the authors report a simple method to measure the cell-cell interaction using adherent cells on two opposing coverslips. Several microscopic tools such as bright field, confocal, STED, dSTORM, SOFI were used to image the interfaces between two interacting cells. The authors claim that by changing the orientation of the interfaces from perpendicular to parallel to the coverslips, it allows imaging the cell-cell interfaces at a faster speed and higher resolution. However, as mentioned by the authors, the optical resolution in the z direction (perpendicular to coverslip) is the worse so is the sectioning capability. In such configuration, the imaging plane is too thick compared with the cell-cell interfaces such as immune synapse (IS). It is very difficult to identify which structures in the images are located within IS. In addition, the cell-cell interface is not a fixed plane. As seen from figure 3, the cell membranes are very dynamic and there are a lot of membrane raffles. Therefore, three-dimensional imaging is needed to resolve the structures at the interfaces. It seems to us that the authors is unaware of the recent development of lightsheet microscopy, which can provide very high resolution three-dimensional images at high speed. In one example, the lightsheet microscopy has been utilized to visualize the immune synapse in three dimension with a very high spatiotemporal resolution (Immunity 2015, 42, 864). In addition, to demonstrate the reported technique a better alternative to the existing methods, we would expect that the authors should provide 1) well-defined interfaces for the observation of cell-cell interactions 2) allowing observation of multiple cell-cell interactions on the same device, and 3) live imaging of cell-cell interactions with detail structures. In our opinion, the authors fail to deliver these important features in their technique.

Major issues:

1. The authors claim that by realigning the cell-cell interfaces parallel to the coverslip, it will eliminate the need of scanning in the z-direction. Therefore, the imaging speed at the interfaces can be increased. However, from figure 2B it can be clearly seen that the interfaces between two cells are not planar. Therefore, the detailed structures cannot be obtained by imaging a single plane. 3D imaging is necessary. Since the resolution in the z-direction is the worse for most of the optical microscopy, the imaging plane contains signals, not from the cell-cell interfaces. For example, in figure 2A, even with super-resolution microscopy, STED, the signals away from the focal plane still contribute significantly to the image. In z=0 bottom of the white box, we can see four bright spots, which can still be visualized at z=1um. Therefore, it is very difficult to identify which structures are located at the interfaces.
2. The authors are not so familiar with the super-resolution microscopy. They don’t know how to define resolution in the image. In figure 2C, they just draw two lines across some features in the images and claimed a small dip in the line profile as their imaging resolution. In such a noisy image, it is hard to believe that 30 nm resolution has been achieved. The resolution of the image depends on many factors including the number of the detected photons, labeling density and the structures in the image. A more appropriate approach is to calculate the Fourier ring correlation. The same argument also applies for the single-molecule localization microscopy. The number of the detected photons only determines the uncertainty in fitting, not the resolution.
3. Figure 4 is the only time-lapse image at the cell-cell interfaces. However, there is no detail description on how these images are obtained. From the progression of the cell-cell interaction, we would guess that the CD8+ cell is coming from left. The interface is perpendicular to the imaging plane, which is different from the method proposed by the authors. These images provide a better view of cell-cell interaction as compared with Figure 3. The authors claimed that they used dSTROM to obtain these images. However, we don’t see the advantage of super-resolution imaging in these applications. There are no fine structures needed to be resolved. If dSTORM is used, how do the authors distinguish the out of focus signals? Do they use optical astigmatism?
4. At the immune synapse, there are a lot of protein-protein interactions. The authors applied several super-resolution techniques to image the cells. However, in many cases, non-specific dyes are used. The authors should label specific proteins and study the protein-protein interactions at the cell-cell

interfaces.

Reviewer #2 (Remarks to the Author):

The manuscript "High and super-resolution imaging of cell-cell interfaces between opposing surfaces" by Sajman, Razvag, et al. presents a simple method to establish cell-cell interfaces for optical microscopy perpendicular to the optical axis. This geometry enables to capture the cell-cell interface in the x/y plane, which provides improved resolution compared to a geometry where the interaction plane is parallel to the optical axis (along z). The sample preparation is well characterized and easy to implement. Fundamentally, I regard this work as suitable for publication in Communications Biology.

My key concern is that the manuscript employs a plethora of super-resolution techniques to the studied systems without rigorously quantifying their performance. Instead, much attention is paid to emphasizing the versatility and superiority of the "opposing surfaces" technique, e.g. in Table 1. For example, only localization accuracies are given for SMLM in Figure S4, but no values for signal, background, number of localizations, or the chosen methods to visualize the localization data (such as 2D histograms). The lack of detail is especially regrettable as it draws the attention of the reader away from the key invention of the work, i.e. the sandwich sample preparation.

I strongly recommend rewriting all sections of the paper that are concerned with microscopy both in the main text and the methods, including exact performance quantification and detailed information on the data shown for all the applied super-resolution microscopy techniques. Some specific suggestions:

• Please include central microscope settings for all setups (detector, camera pixel size, filters, ...)

• On page 11, the authors report that they were able to "resolve features as small as 30 nm" using STED, which, if this translates to an actual resolution, would be an exceptional value for STED microscopy in a biological sample. But as no rigorous PSF quantification of the STED PSF is reported, it is unclear to the reader how to interpret this value. From the intensity trace shown, this feature seems rather like a random fluctuation.

• Could the authors provide more details on Figure 4? It seems like a full two-color dSTORM-based SMLM reconstruction was achieved every 15 seconds, which is exceptional. For example, how many localization were recorded? What is the localization precision?

• The axes shown in Figure 4 top are very confusing. Is this a temporally resolved 3D stack? Or a 2D projection of a 3D stack with 3 microns width? Why is the zoom-in tilted?

• The post-processing methods SOFI and SRRF require several parameters to be set manually. Please report the exact settings.

• The key argument for the geometry the authors chose is improved resolution compared to a geometry where the cell-cell interface is parallel to the optical axis. However, the authors do not compare their method rigorously to such a geometry. Certainly, the imaging in the x/y plane typically exhibits improved resolution. However, for a paper that describes a mainly methodological improvement, benchmarking against the standard is crucial. The authors should include comparisons to the "unfavorable case".

In addition, I would suggest that the authors comment on the bigger picture of their method compared to already established strategies:

• Several methods exist to study cell-cell interactions with optical microscopy. Specifically, the immunosynapse has already been investigated with super-resolution microscopy and single-molecule tracking multiple times in great detail. Could the authors comment on the specific advantage of their approach?

• Many cells involved in biologically relevant synapse formation, for example NK cells, are not adherent and, if forced to adhere, exhibit different behavior. Is this a limitation of the proposed method?

In summary, the method presented by the author seems versatile and robust, but even though many flavors of microscopy are applied to the created samples, the lack of quantitative details regarding the applied methods makes it hard to assess the results. Reporting quantitative parameters and settings is mandatory and will significantly improve the manuscript.

Reviewers' comments:

Reviewer #1 (Remarks to the Author):

In this manuscript, the authors report a simple method to measure the cell-cell interaction using adherent cells on two opposing coverslips. Several microscopic tools such as bright field, confocal, STED, dSTORM, SOFI were used to image the interfaces between two interacting cells. The authors claim that by changing the orientation of the interfaces from perpendicular to parallel to the coverslips, it allows imaging the cell-cell interfaces at a faster speed and higher resolution. However, as mentioned by the authors, the optical resolution in the z direction (perpendicular to coverslip) is the worse so is the sectioning capability. In such configuration, the imaging plane is too thick compared with the cell-cell interfaces such as immune synapse (IS). It is very difficult to identify which structures in the images are located within IS.

We agree with the reviewer that the interface between cells is much thinner than the sectioning of our microscope (and most others) in the z-direction (of ~700nm). An ideally flat immune synapse would span <200nm (including membranes, extracellular and intracellular membrane proteins, and related protein complexes. Membrane protrusion and ruffling and cell curvature can further significantly increase this size of cross-section of interest.

Still, we would like to clarify that we do not intend to restrict our measurements to a single cross-section, but rather limit the z-range for which such cross-sectioning is needed for optimal capturing of the immune synapse.

The main advantage of our approach is in gaining much higher spatial resolution in the x-y plane. We achieve this in a way that is widely compatible with common superresolution microscopy techniques. This advantage can now be evaluated by comparing our cell-on-cell imaging (e.g. Fig. 3 via dSTORM) with the more traditional approach of imaging cell conjugates side-by-side (Fig. S6 A,B via dSTORM).

Figure S6

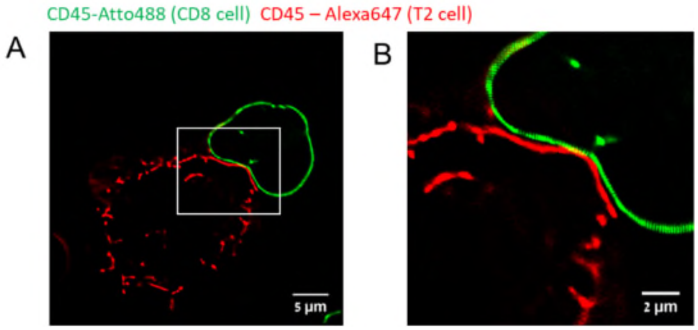


"**Fig S6. Imaging of side-by-side cellular interfaces**

(A) dSTORM imaging of a synapse formed between cell-cell conjugates as they form side by side. The CD8$^{+}$ T cells were stained with CD45 and Atto488 (green) and T2 cells were stained with CD45-Alexa647 (red). On right, a zoom in on the synapse between two cells. (B) Bright-field (BF) and dSTORM imaging (merged on left) of an interface of a cell and a 20µm silicon bead, as they

adhere side by side. CD8$^+$ T cells were stained with DIO (green) and silicon beads were stained with αCD3ε-Alexa647 (red). On right, zoom in on the interface between the bead and the cells."

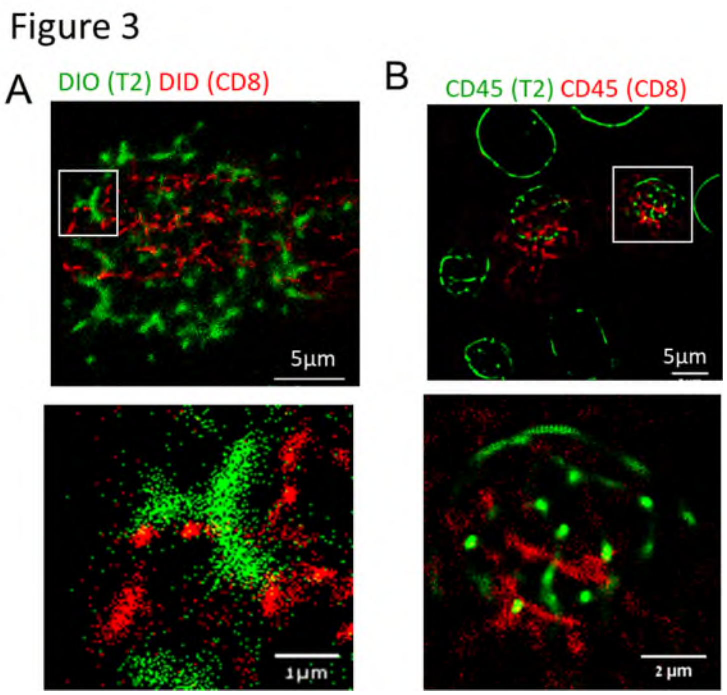


**"Fig. 3. Molecular organization and interactions at the IS**
(A-E) dSTORM images of cell conjugates of CD8+ T cells with T2 cells loaded with the activating peptide NY-ESO-1. (A) The PM of the CD8+ T cells was stained with DiD (red) and the PM of the T2 cells was stained with DiO (green). A representative cell (top) and zoom image (bottom) are shown. (B) The CD8+ T (red) and T2 (green) cells were both stained for CD45 (with Alexa647 and Atto488 secondary antibodies, respectively). A representative field (top) and zoom image of a single conjugate (bottom) are shown."

In addition, the cell-cell interface is not a fixed plane. As seen from figure 3, the cell membranes are very dynamic and there are a lot of membrane raffles. Therefore, three-dimensional imaging is needed to resolve the structures at the interfaces. It seems to us that the authors is unaware of the recent development of lightsheet microscopy, which can provide very high resolution three-dimensional images at high speed. In one example, the lightsheet microscopy has been utilized to visualize the immune synapse in three dimensions with a very high spatiotemporal resolution (Immunity 2015, 42, 864).

We thank the reviewer for this comment and would like to clarify: we have been aware of the LLSM approach when writing our first submission. We have not included it in our table since we focused on methods for re-orientation of the immune synapse, and it does not strictly provides this capability. Still, we agree with the reviewer that this important method deserves reference and discussion, which we have now added (as below):

Pros: LLSM is very fast and provides 3D images with ~230x230x370nm$^3$ resolution of whole cells (and cell-cell conjugates) within seconds.

Cons: LLSM is a relatively complicated system that employs Bessel beams for illumination, while illumination and imaging use two different objectives that are carefully aligned. Thus, this method is relatively expensive, hard to calibrate and operate. Importantly, LLSM resolution is still much lower than our approach, achieving images in single molecule detail with spatial resolution <34nm. Moreover, LLSM is not open for integration with multiple imaging modalities, as we demonstrate for our method in this study.

We have now added the following paragraph to the discussion:

"Recently, the introduction of lattice light sheet microscopy (LLSM)[43] to the imaging of cell-cell conjugates has allowed very fast imaging of the evolving interface (1.3sec for 130 z-cross-sections)[44]. The lateral spatial resolution is 230nm and the z-resolution is 370nm[43]. Thus, it improves the z-sectioning capability, yet is still far from the resolution that we show for features within the imaged interfaces. The clear advantage of the LLSM technique over our approach is its very fast time resolution of the entire cell [45] or of the cell-cell conjugate [44].

We have also added a column related to LLSM to Table 1 (in which the rest of the approaches are compared).

In addition, to demonstrate the reported technique a better alternative to the existing methods, we would expect that the authors should provide

1) well-defined interfaces for the observation of cell-cell interactions

In response to this comment, we have used 20 micron beads as well-defined surfaces for forming a functional interface with T cells. Specifically, the beads were coated with anti-CD3ε antibody labeled with Alexa647. We then performed dSTORM imaging between the beads and cells highlighted with DIO or PALM imaging of cells that expressed TCRζ-Dronpa (together with dSTORM imaging of the beads). The results are shown in new Fig. 4A,B (see below). The experiments and related results are detailed as follows:

"The cell-cell interfaces formed using our approach are often irregular and may be of interest at a range of focal planes. Thus, we further wanted to test our approach and its imaging performance for more regular interfaces. For that, we conjugated 20μm spheres adhered to a top coverslip with T cells adhered to the bottom coverslip (Fig. 4A,B). The spheres were labelled with αCD3ε-Alexa647 antibody, and could be clearly observed by both bright-filed (BF) imaging and by dSTORM. In one set of experiments, $CD8^+$ T cells were highlighted with the non-specific PM dye DIO (Fig. 4A). Alternatively, we conducted photo-activated localization microscopy (PALM) of $CD4^+$ T cells, expressing TCRζ-Dronpa [23] (Fig. 4B). Interestingly, the TCRζ-Dronpa molecules co-localized with the αCD3ε antibodies, suggesting that they could specifically bind and aggregate them across the surface of the beads. Thus, we show that our imaging approach can also combine dSTORM and PALM for 2 color SMLM."

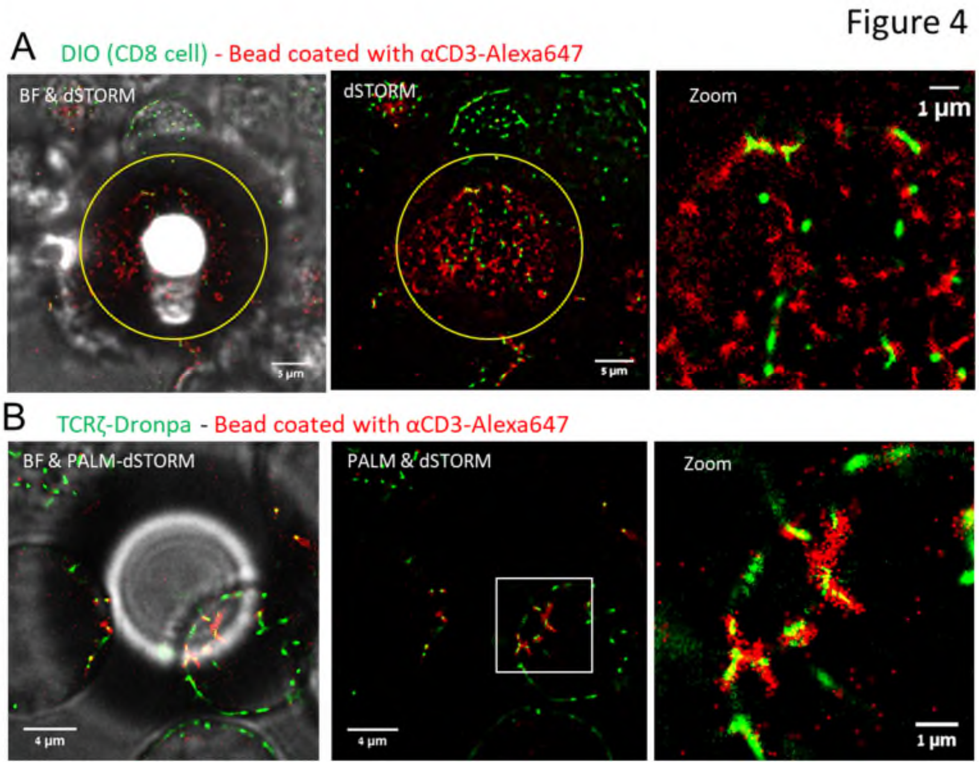


“**Fig. 4. Molecular organization at cell-bead interfaces and using multiple super-resolution reconstruction techniques**

(A) **The interface between 20µm silicon beads and CD8$^+$ T cells**. The CD8$^+$ T cells were stained with DIO (membrane dye) and adhered to the bottom coverslip. 20µm silicon beads (highlighted diameter in yellow circle) were coated with αCD3ε-Alexa647, and adhered to the top coverslip. A representative cell-bead interface is shown Bright field of the cell-bead interface, merged with the two fluorescence channels (left panel) Fluorescence imaging of DIO (green) and αCD3ε (red) (middle panel) Zoom in on the cell-bead interface (right panel).

(B) **The interface between 20µm silicon beads and Jurkat E6.1 T cells**. Jurkat E6.1 (CD4$^+$ T) cells stably expressing TCRζ-Dronpa adhered to the bottom of coverslip. On top, 20µm silicon beads coated with αCD3ε-Alexa647. A representative cell-bead interface is shown. Bright field merged with fluorescence (left panel). Fluorescence of Dronpa (green) and CD3ε (red) (middle panel). Zoom in on the cell-bead interface (right panel)."

2) allowing observation of multiple cell-cell interactions on the same device, and

We would like to clarify that our approach allows for imaging multiple cell-cell interactions on the same devise. For obtaining simultaneous high and super-resolution imaging of multiple cell conjugates, these conjugates could be captured in the same field of view (e.g. see Movie M2; Still, this typically requires relatively denser samples). Alternatively, one could screen many such conjugates first in low resolution, and then with high or super-resolution with a much smaller FOV. This latter procedure is demonstrated by moving from very large FOVs (tiled together) in Fig. S1 and subsequent focusing on specific FOVs and conjugates, as demonstrated in Fig. 1B and in the rest of the manuscript.

3) live imaging of cell-cell interactions with detail structures.

We would like to stress that most of our imaging was conducted on live cells (all, except Fig. 2). The dynamics of structures are shown in Fig. 5. To better illustrate this point we have now added two movies (Movies M1,M2). These movies show the interface of $CD8^+$ T cells and T2 cells as they form the conjugate side-by-side (Movie M1), or one on top of the other (Movie M2; The middle cell conjugate is the one shown in Fig. 4C-F). While only the PM of the cells is visible in Movie M1, a reach nanoscale patterning is visible in movie M2. Single frames from movies M1 and M2 are shown below (for convenience).

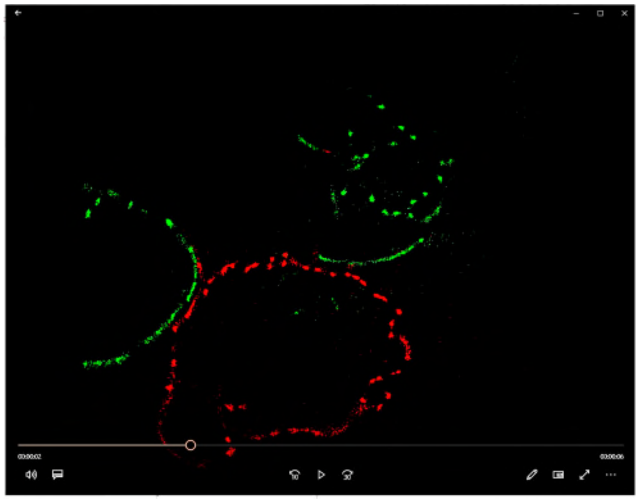

**"Movie M1. dSTORM imaging of live cell-cell conjugates that form side-by-side**
dSTORM imaging of a synapse formed between cell-cell conjugates as they form side by side. The $CD8^+$ T cells were stained with CD45-Atto488 (green) and T2 cells were stained with CD45-Alexa647 (red). See methods for details on rendering of the movie."

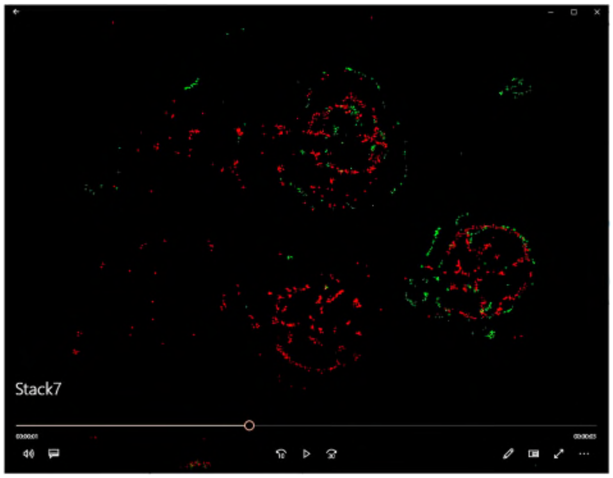


**"Movie M2. dSTORM imaging of live cell-cell conjugates that form one on top of the other**
dSTORM imaging of synapses formed between cell-cell conjugates as they one on top of the other. The $CD8^+$ T cells were stained with DID (red) and T2 cells were stained with DIO (green). See methods for details on rendering of the movie."

In our opinion, the authors fail to deliver these important features in their technique.
As discussed and shown above, our approach provides a simple and accessible means for delivering the important feature specified by the reviewer, including live cell imaging, multiple cell-cell interfaces, and demonstration with well-defined interfaces.

Major issues:
1. The authors claim that by realigning the cell-cell interfaces parallel to the coverslip, it will eliminate the need of scanning in the z-direction.
We would like to clarify this point. Our intention is not to eliminate z-scanning altogether and in all cases. In fact, we do employ z-sectioning and scanning in multiple cases in this study, e.g. in Fig. 1B, 2, in 4A-F (not shown), 5, S1, S2C, D, S3, S4, S6 (not shown). However, our scanning now typically aims to either quickly identify the plane(s) of interest where the cell-cell interface occurs, or to create a 3D image of this interface. Notably, the number of the cross-sections that are needed for capturing the cell-cell interface is clearly smaller than when cells are captured side-by-side, and with much less details (as shown in Fig. S6A,B for comparison).

Therefore, the imaging speed at the interfaces can be increased. However, from figure 2B it can be clearly seen that the interfaces between two cells are not planar. Therefore, the detailed structures cannot be obtained by imaging a single plane. 3D imaging is necessary. Since the resolution in the z-direction is the worse for most of the optical microscopy, the imaging plane contains signals, not from the cell-cell interfaces. For example, in figure 2A, even with super-resolution microscopy, STED, the signals away from the focal plane still contribute significantly to the image. In z=0 bottom of the white box, we can see four bright spots, which can still be visualized at z=1um. Therefore, it is very difficult to identify which structures are located at the interfaces.
We agree with the reviewer that the cross-sectioning on our assays does not provide super-resolution and is limited by the Raleigh distance to ~700nm. This holds also for the confocal/STED imaging in Fig. 2A. The bright features in the zoom there likely reside in between the imaged planes and are sufficiently bright to show in 2 sections (z=0,+1 microns). It is also possible that the membrane stain of the T2 cell (in green) highlighted intracellular vesicles.
We also agree with the reviewer that cell-cell interfaces are typically not flat. Still, as mentioned before, we do not aim to eliminate z-sectioning altogether, but to reduce the number of such sections and improve the resolutions of lateral features of interest, as compared to side-by-side imaging of cell-cell interfaces.

2. The authors are not so familiar with the super-resolution microscopy. They don't know how to define resolution in the image. In figure 2C, they just draw two lines across some features in the images and claimed a small dip in the line profile as their imaging resolution. In such a noisy image, it is hard to believe that 30 nm resolution has been achieved. The resolution of the image depends on many factors including the number of the detected photons, labeling density and the structures in the image. A more appropriate approach is to calculate the Fourier ring correlation. The same argument also applies for the single-molecule localization microscopy. The number of the detected photons only determines the uncertainty in fitting, not the resolution.

We thank the reviewer for this important comment. As a response, we have now added multiple data that quantitatively show the improved resolution using our approach. The resolution estimation using Fourier Ring Correlation was 34nm for dSTORM images, and ~160nm for SRRF (FRC; Fig. S4E,F; see methods for FRC estimations). Localization parameters and uncertainties are provided for the used fluorophores and imaging configurations (Fig. S5). STED imaging shows resolution of 73.5nm, based on PSF through calibration using 40nm fluorescent beads (Fig.S4A-D). We now describe this data and analyses in the main text as follows:

"Our SMLM imaging resulted in distributions of localisation parameters and uncertainties that were overall similar for cell-cell conjugates forming side-by-side (Fig. S5A,B) panels) and for cells one on top of the other (Fig. S5C,D). Under all conditions, we note a bimodal histogram of localization uncertainties, often with a primary mode at 10-20nm, and a shoulder (or a secondary mode) at 30-40nm (see SI for details on imaging and analyses). Notably, the related resolution is comparable to the resolution obtained by total internal reflection fluorescence (TIRF) imaging at the interface of the cells with functionalized coverslips (e.g. [37]). Fourier-Ring Correlation analysis of our dSTORM imaging yielded an effective resolution of < 34nm (Fig. S4E; see details regarding FRC analyses in Methods)."

Figure S4

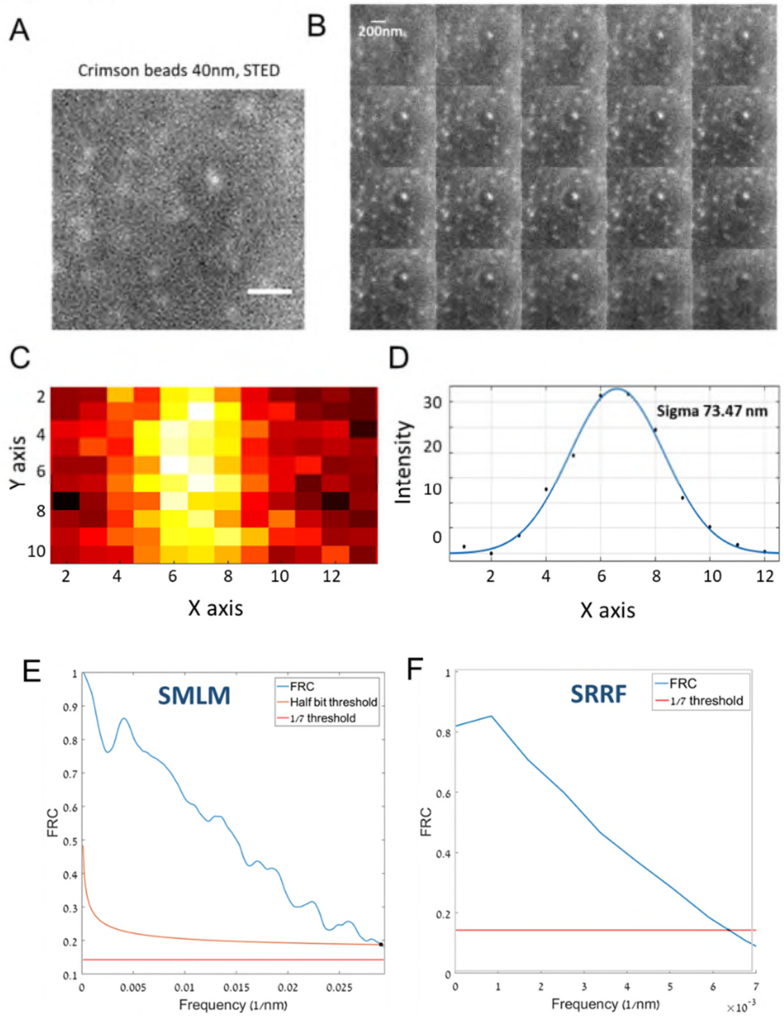

**"Fig. S4. Resolution of STED, SMLM and SRRF**

(A) Crimson beads (40 nm size) imaged by STED in xy projections. (B) Z stacks of the same image in panel (A). (C) Projection of intensity of a single Crimson bead along the x-z plane. (D) Intensity distribution of a single Crimson bead along the Z-axis, showing the width of the convolution between the STED PSF and the bead.

(E) Fourier ring correlation (FRC) of the SMLM reconstruction in Fig. 4F. Blue line: FRC of the experimental data, Orange line: Half-bit threshold estimate (with 34nm cut-off point); Red line: 1/7 threshold. (F) FRC of the SRRF reconstruction presented in Fig. 4E, as compared with the SMLM image, yielding a SRRF resolution estimate of 160nm."

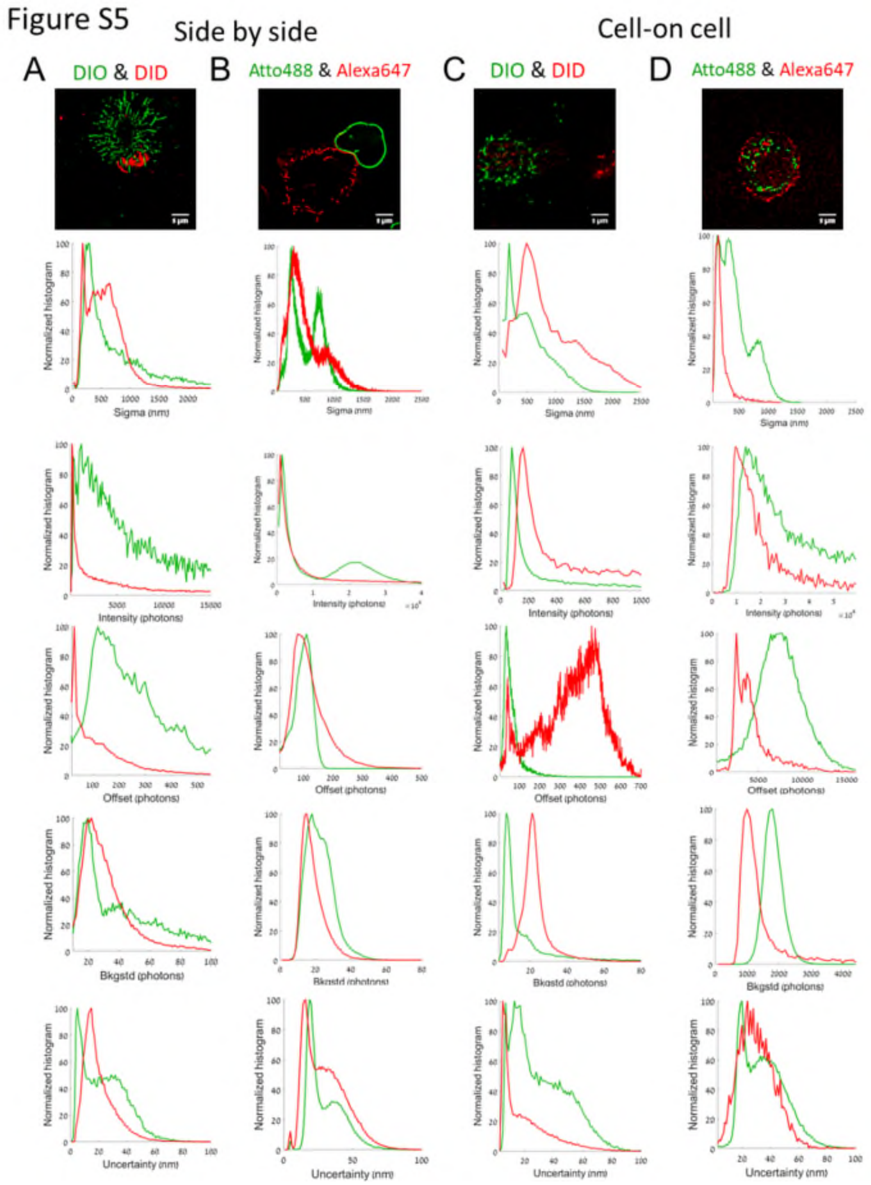


**"Fig. S5. Localization accuracy of SMLM when imaging on opposing coverslips**

Data of ThunderSTORM analysis of dSTORM imaging of CD8$^+$ and T2 cell conjugates. (A,B) Conjugates forming side-by-side interfaces. Cells were labelled with either (A) DIO and DID, or (B) Alexa647 and Atto488. (C,D) Conjugates forming cell-on-cell interfaces. dSTORM images of the cellular interfaces are shown. Again, cells were labelled with either (C) DIO and DID, or (D) Alexa647 and Atto488. For each panel, histograms are shown of different parameters include: Sigma [nm]: standard deviation of the Gaussian fitted to the peak. Intensity [photons]: integrated photons number under the peak (value used to calculate the uncertainty in the Webb/Mortensen formulas).Offset [photons]: baseline of the peak (background absolute value). Bkgstd [photon]: standard deviation of the background (used to calculate the uncertainty in the Webb/Mortensen formulas). Uncertainty_[nm]: standard deviation of the lateral localization uncertainty (used to draw the width of each localization when using Gaussian rendering). The number of Green/Red localizations for each image was: (A) 21,738; 61,631 (Red), (B) 103,835; 150,626, (C) 31,427; 49,097, (D) 55,911; 4,695."

3. Figure 4 is the only time-lapse image at the cell-cell interfaces. However, there is no detail description on how these images are obtained. From the progression of the cell-cell interaction, we would guess that the CD8+ cell is coming from left. The interface is perpendicular to the imaging plane, which is different from the method proposed by the authors. These images provide a better view of cell-cell interaction as compared with Figure 3. The authors claimed that they used dSTROM to obtain these images. However, we don't see the advantage of super-resolution imaging in these applications. There are no fine structures needed to be resolved. If dSTORM is used, how do the authors distinguish the out of focus signals? Do they use optical astigmatism?
To clarify, the imaging of Fig. 5 (formerly Fig. 4) was conducted using confocal microscopy, as described in the Methods (section on Confocal/STED microscopy). In this time-lapse images, the cell is indeed approaching from left but forms a *partially* parallel interface, which is imaged.
Specifically, there are membrane protrusions (lamellae) from the side of the CD8$^+$ T cells, which are sent out toward the T2 cell upon cell engagement. This feature is highlighted with an arrow at the bottom zoom image. Furthermore, CD45 is known to form clusters at the PM of T cells, as can be clearly seen in the different images (cross-section of the CD8$^+$ T cell lower-side membrane). The cell-cell interface is clearly not perpendicular at this stage. The cells are at different heights, and the cell-cell interface is at ~45deg (red features can be seen on top of green features).

4. At the immune synapse, there are a lot of protein-protein interactions. The authors applied several super-resolution techniques to image the cells. However, in many cases, non-specific dyes are used. The authors should label specific proteins and study the protein-protein interactions at the cell-cell interfaces.
We thank the reviewer for this comment. We have now used our approach to image multiple protein-protein interactions across the immune synapse. Specifically, we visualized interactions of the integrins ICAM1 and LFA1α (already shown in our previous submission). Newly imaged interactions include CD28-CD80 and CTLA4-CD80. All interactions were imaged via dSTORM (See updated Fig. 3). The added data is described in the main text as follows:
"Additional protein-protein interaction occur between CD80 on APCs (here, T2) and both CD28 and CTLA4 on T cells. CD28 is a co-stimulatory molecule that augments TCR activation [34]. In contrast, CTLA4 engagement by CD80 inhibits TCR-dependent activation [35,36]. Here, we

observed co-clustering of CD80 and CD28 (Fig. 3D), or with CTLA4 (Fig. 3E) across the IS in some cell-conjugates, but not in all (compare zoom regions of individual conjugates in each panel).

Figure 3

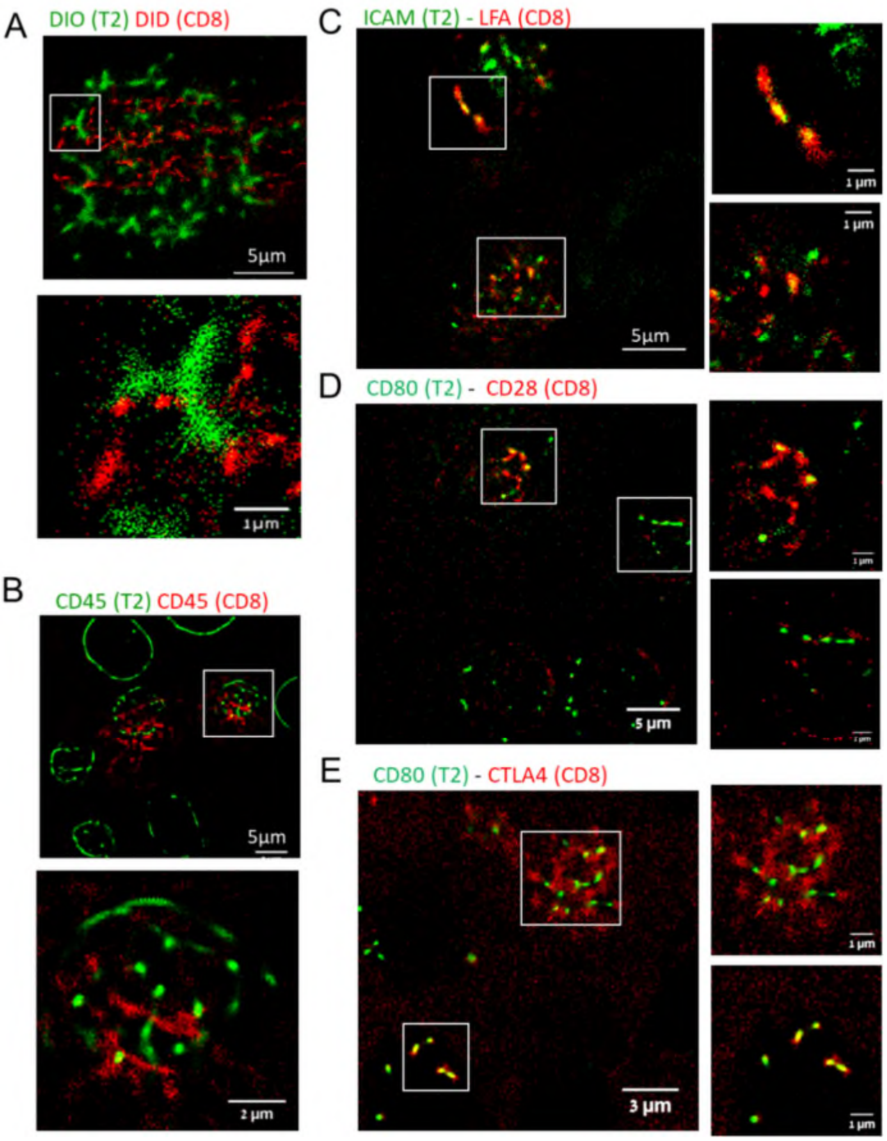


**"Fig. 3. Molecular organization and interactions at the IS**
(A-E) dSTORM images of cell conjugates of CD8$^+$ T cells with T2 cells loaded with the activating peptide NY-ESO-1. (A) The PM of the CD8$^+$ T cells was stained with DiD (red) and the PM of the T2 cells was stained with DiO (green). A representative cell (top) and zoom image (bottom) are shown. (B) The CD8$^+$ T (red) and T2 (green) cells were both stained for CD45. A representative field (top) and zoom image of a single conjugate (bottom) are shown. (C) The CD8$^+$ T cells (red) were stained for LFA1a, and T2 (green) cells were stained for ICAM1. A representative field (left) and two zoom images of single conjugates (right) are shown. (D) The CD8$^+$ T cells (red) were stained for CD28, and T2 (green) cells were stained for CD80. A representative field (left) and two zoom images of single conjugates (right) are shown. (E) The CD8$^+$ T cells (red) were stained for

CTLA4, and T2 (green) cells were stained for CD80. A representative field (left) and two zoom images of single conjugates (right) are shown."

Reviewer #2 (Remarks to the Author):

The manuscript "High and super-resolution imaging of cell-cell interfaces between opposing surfaces" by Sajman, Razvag, et al. presents a simple method to establish cell-cell interfaces for optical microscopy perpendicular to the optical axis. This geometry enables to capture the cell-cell interface in the x/y plane, which provides improved resolution compared to a geometry where the interaction plane is parallel to the optical axis (along z). The sample preparation is well characterized and easy to implement. Fundamentally, I regard this work as suitable for publication in Communications Biology.
We thank the reviewer for his interest in our approach and recommendation for publication.

My key concern is that the manuscript employs a plethora of super-resolution techniques to the studied systems without rigorously quantifying their performance. Instead, much attention is paid to emphasizing the versatility and superiority of the "opposing surfaces" technique, e.g. in Table 1. For example, only localization accuracies are given for SMLM in Figure S4, but no values for signal, background, number of localizations, or the chosen methods to visualize the localization data (such as 2D histograms). The lack of detail is especially regrettable as it draws the attention of the reader away from the key invention of the work, i.e. the sandwich sample preparation.
We thank the reviewer for this important comment. In response to this comment (and to related comments by Reviewer 1), we have now added multiple details and analyses that rigorously quantify the performance of our imaging modalities. Specifically, we provide the following:
* For dSTORM we now provide histograms for parameters related to the signal, background, and we specify the number of localizations (specific localization parameters incl. Sigma, Intensity, Offset, Background and Uncertainty). Histograms of the resultant parameters of the reconstruction process are shown in new Fig. S5 (shown above in response to the 2$^{nd}$ comment of reviewer 1).The chosen method for dSTORM reconstruction was ThunderSTORM version 1.3. All relevant parameters are now specified in the Methods.
* For STED we now provide the PSF (see in response to a comment below).
* For dSTORM and SRRF we further provide the FRC curves for determination of their spatial resolution when imaging the upper cell in the interface (for being conservative). We get dSTORM resolution of <34nm and SRRF resolution of ~160nm. Details regarding the analyses is provided in the Methods. Fig. S7 shows the relative resolution enhancement and background elimination capabilities of (2$^{nd}$ order) SOFI.

I strongly recommend rewriting all sections of the paper that are concerned with microscopy both in the main text and the methods, including exact performance quantification and detailed information on the data shown for all the applied super-resolution microscopy techniques.
The data above, including performance quantification, details related to the super-resolution imaging and reconstruction techniques, are now elaborated in multiple places in the text (see blue

text in the resubmitted manuscript, in the Results, Methods, and in the Supplementary Figures and legends).

Some specific suggestions:

• Please include central microscope settings for all setups (detector, camera pixel size, filters, ...).
We have now included the requested details on the wide-filed TIRF microscope and confocal/STED microscope in the Methods (see blue text there).

• On page 11, the authors report that they were able to "resolve features as small as 30 nm" using STED, which, if this translates to an actual resolution, would be an exceptional value for STED microscopy in a biological sample. But as no rigorous PSF quantification of the STED PSF is reported, it is unclear to the reader how to interpret this value. From the intensity trace shown, this feature seems rather like a random fluctuation.
Following the reviewer comment, we performed PSF quantitation of STED images. Fig. S4 shows PSF with sigma of 73.5nm. Accordingly, we write: "The point-spread-function (PSF) of the STED microscope was tested using 40nm crimson beads (Fig. S4A,B) and had a width (sigma, in the lateral plane) of 73.5nm (Fig. S4C,D). This implies a STED resolution of ~60nm for a point emitter."

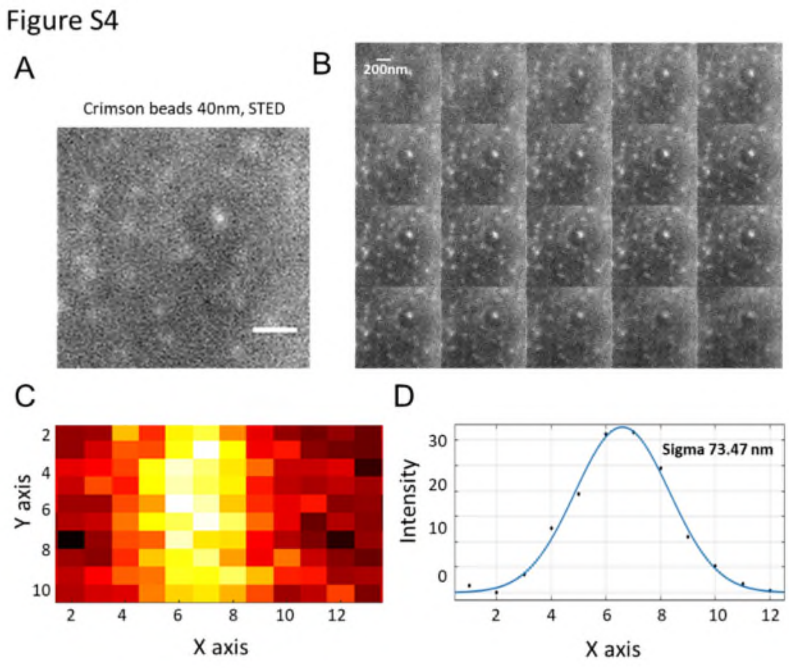


**"Fig. S4 Resolution of STED, SMLM and SRRF**

(A) Crimson beads (40 nm size) imaged by STED in xy projections. (B) Z stacks of the same image in panel (A). (C) Projection of intensity of a single Crimson bead along the x-z plane. (D) Intensity distribution of a single Crimson bead along the Z-axis, showing the width of the convolution between the STED PSF and the bead."

• Could the authors provide more details on Figure 4? It seems like a full two-color dSTORM-based SMLM reconstruction was achieved every 15 seconds, which is exceptional. For example, how many localization were recorded? What is the localization precision?

We thank the reviewer for this comment. Indeed, live cell two-color SMLM is non-trivial and requires fast imaging of bright fluorophores and careful reconstruction of image stacks. We now provide more details on this imaging, which is demonstrated in new Movies M1 and M2. Localization statistics of live-cell 2-colour dSTORM imaging are detailed in new Fig. S5. Imaging is shown there form both cells forming interfaces side-by-side (panels A,B) or one on top of the other (panels C,D).

Otherwise, we apologize for a previous confusion in writing. Fig. 5 (former Fig. 4) was captured using a z stack of confocal x-y scan. In this type of measurements, temporal resolution of a few seconds is achievable.

• The axes shown in Figure 4 top are very confusing. Is this a temporally resolved 3D stack? Or a 2D projection of a 3D stack with 3 microns width? Why is the zoom-in tilted?

We apologize for any possible confusion and would like to clarify: the images in Fig. 5 (previously Fig. 4) are 3D projections. The 2D images are projections of 3 micron slices. We now further clarify this in the related text and legend of this figure.

We chose to present the zoom images as tilted to better highlight the 3D nature of the interface. In this interface the upper red ($CD8^+$) cell approaches the bottom green (T2) cell from its upper left side. Thus, an interface is formed with a significant horizontal overlap between the cells.

• The post-processing methods SOFI and SRRF require several parameters to be set manually. Please report the exact settings.

The processing parameters of SOFI and SRRF are now described in the Methods, as follows:

"SRRF and SOFI reconstruction

We acquired the SRRF images using the published software [40] with the following parameters: Ring = 0.10, radial magnification = 10, frames per point = 5. Second-order SOFI reconstruction was performed using the Matlab SOFI version provided by [56] with the default parameters."

• The key argument for the geometry the authors chose is improved resolution compared to a geometry where the cell-cell interface is parallel to the optical axis. However, the authors do not compare their method rigorously to such a geometry. Certainly, the imaging in the x/y plane typically exhibits improved resolution. However, for a paper that describes a mainly methodological improvement, benchmarking against the standard is crucial. The authors should include comparisons to the "unfavorable case".

We performed experiments to directly compare the performance of our imaging approach relative to standard (or more common) approaches. For that, we imaged cells that formed an interface side-by-side (See Fig. S6A,B). The images can now be readily compared with images acquired using our approach using the cell-on-cell configuration (e.g. Fig. 3A,B).

Figure S6

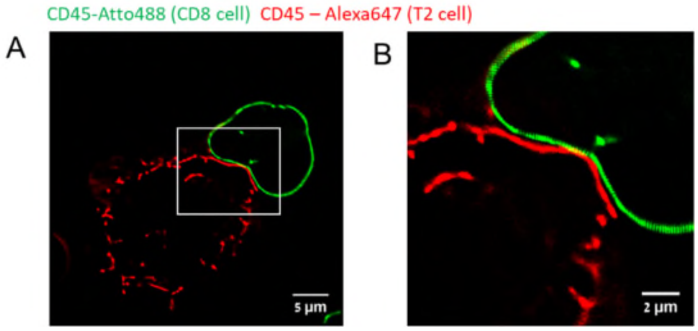


**"Fig S6. Imaging of side-by-side cellular interfaces**
(A) dSTORM imaging of a synapse formed between cell-cell conjugates as they form side by side. The CD8$^+$ T cells were stained with CD45 and Atto488 (green) and T2 cells were stained with CD45-Alexa647 (red). On right, a zoom in on the synapse between two cells. (B) Bright-field (BF) and dSTORM imaging (merged on left) of an interface of a cell and a 20µm silicon bead, as they adhere side by side. CD8$^+$ T cells were stained with DIO (green) and silicon beads were stained with αCD3ε-Alexa647 (red). On right, zoom in on the interface between the bead and the cells."

Figure 3

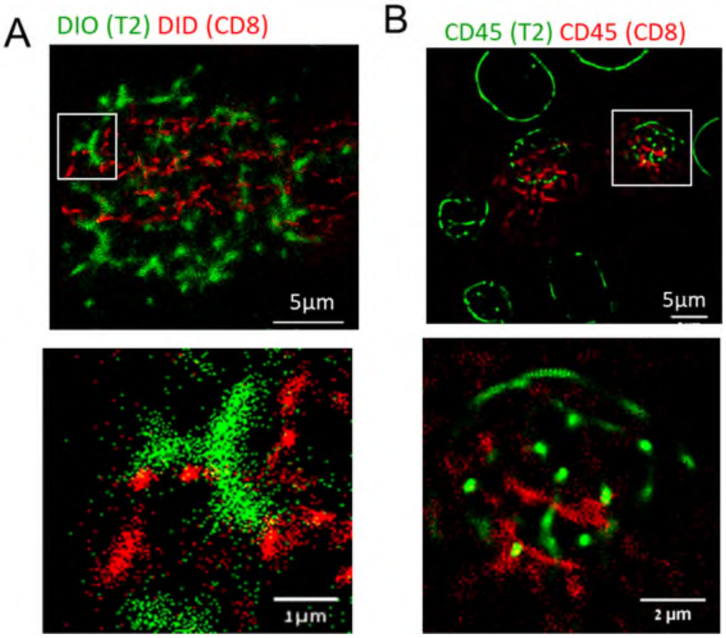


**"Fig. 3. Molecular organization and interactions at the IS**
(A-E) dSTORM images of cell conjugates of CD8+ T cells with T2 cells loaded with the activating peptide NY-ESO-1. (A) The PM of the CD8+ T cells was stained with DiD (red) and the PM of the T2 cells was stained with DiO (green). A representative cell (top) and zoom image (bottom) are shown. (B) The CD8+ T (red) and T2 (green) cells were both stained for CD45 (with Alexa647 and Atto488 secondary antibodies, respectively). A representative field (top) and zoom image of a single conjugate (bottom) are shown."

We also imaged cells as they adhered to 20 micron beads (beads-on-cells, and also side-by-side). These beads provided a more defined surface for interface formation of the cells. Specifically, the beads were coated with anti-CD3ε antibody labeled with Alexa647. We then performed dSTORM imaging between the beads and cells highlighted with DIO or PALM imaging of cells that expressed TCR-ζ-Dronpa (together with dSTORM imaging of the beads). The results are shown in new Fig. 4A,B (see below). Fig. S6B show side-by-side interface of the cells and beads.

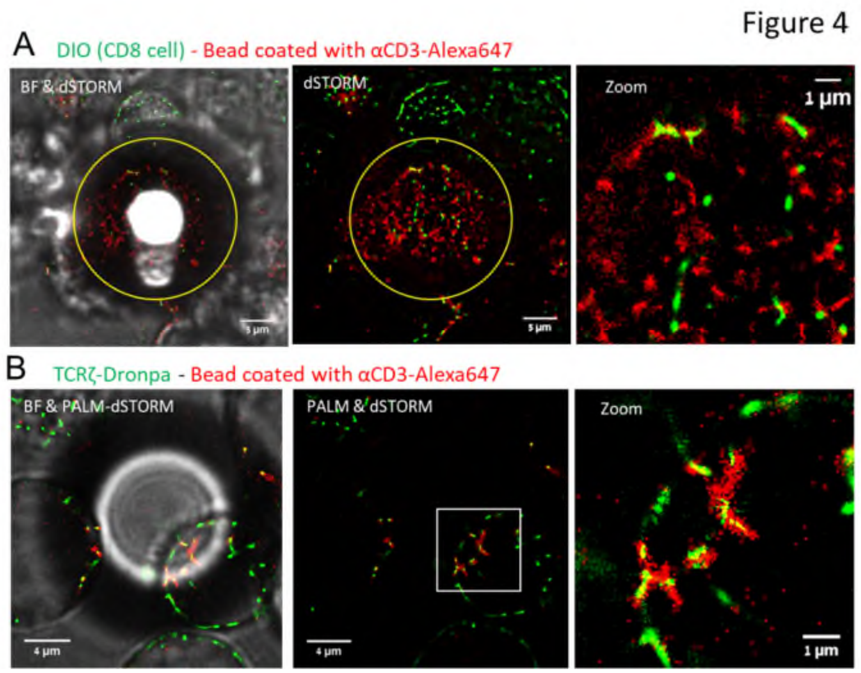


**"Fig. 4. Molecular organization at cell-bead interfaces and using multiple super-resolution reconstruction techniques**

(A) **The interface between 20μm silicon beads and CD8$^+$ T cells**. The CD8$^+$ T cells were stained with DIO (membrane dye) and adhered to the bottom coverslip. 20μm silicon beads (highlighted diameter in yellow circle) were coated with αCD3ε-Alexa647, and adhered to the top coverslip. A representative cell-bead interface is shown Bright field of the cell-bead interface, merged with the two fluorescence channels (left panel) Fluorescence imaging of DIO (green) and αCD3ε (red) (middle panel) Zoom in on the cell-bead interface (right panel).

(B) **The interface between 20μm silicon beads and Jurkat E6.1 T cells**. Jurkat E6.1 (CD4$^+$ T) cells stably expressing TCRζ-Dronpa adhered to the bottom of coverslip. On top, 20μm silicon beads coated with αCD3ε-Alexa647. A representative cell-bead interface is shown. Bright field merged with fluorescence (left panel). Fluorescence of Dronpa (green) and CD3ε (red) (middle panel). Zoom in on the cell-bead interface (right panel)."

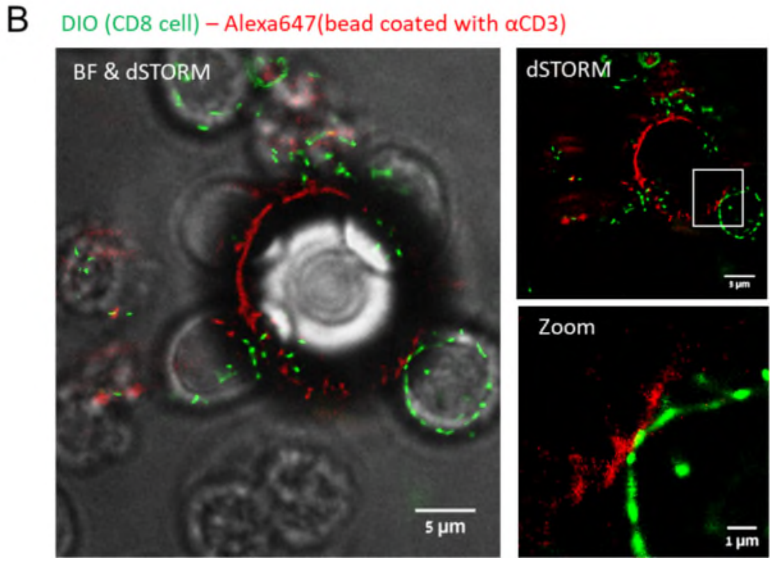


"**Fig S6. Imaging of side-by-side cellular interfaces**
…. (B) Bright-field (BF) and dSTORM imaging (merged on left) of an interface of a cell and a 20µm silicon bead, as they adhere side by side. $CD8^{+}$ T cells were stained with DIO (green) and silicon beads were stained with αCD3ε-Alexa647 (red). On right, zoom in on the interface between the bead and the cells."

In addition, I would suggest that the authors comment on the bigger picture of their method compared to already established strategies:

• Several methods exist to study cell-cell interactions with optical microscopy. Specifically, the immunosynapse has already been investigated with super-resolution microscopy and single-molecule tracking multiple times in great detail. Could the authors comment on the specific advantage of their approach?

Indeed, the immune synapse has been the target of imaging by a wide range of modalities, including diffraction-limited and super-resolution microscopy (see reviews in ref. 6 regarding diffraction-limited microscopy imaging of the IS, and in refs 11,12 for super-resolution of the IS). To best utilize the enhanced resolution of super-resolution microscopy, the researchers typically have turned to artificial mimics of the immune synapse, as T cells adhere and engage a functional surface (e.g. glass-supported lipid bilayer). To visualize the interface between cell-cell conjugates, imaging has been conducted on such conjugates as they form side-by-side with critical loss of details (however, see most recent imaging using lattice light-sheet microscopy; now added to our table and discussion and cited refs 42,43). Alternatively, reorientation of the IS has been achieved using techniques such as optical tweezers and PDMS well-arrays (see Table 1). However, these reorientation efforts have typically not utilized super-resolution imaging since these techniques induce critical complexity and aberrations to the optical system. In contrast, our approach enables super-resolution imaging via a wide range of modalities of optimally reoriented cell-cell conjugates. Thus, we allow easy access to the high and super-resolution imaging of physiological immune-synapses between cell-cell conjugates. Thus, we conclude the introduction as follows:

"We show the compatibility of our approach with multiple diffraction–limited and SRM imaging modalities, including bright-field, confocal and STED imaging, direct STORM (dSTROM), Super-resolution Optical Fluctuation Imaging (SOFI), and Super-Resolution Radial Fluctuations (SRRF). In addition, our technique can be used in large-scale microscopy to allow efficient scanning of multiple interacting cells. We further critically assess the pros and cons of our approach relative to current techniques for cell trapping, highlighting its superiority in stability, simplicity, wide compatibility with various microscopes, and very low background. We expect that these properties of our method will allow the wide-spread use of high-quality diffraction-limited and super-resolution imaging of intercellular interfaces by enabling routine imaging using standard and advanced optical microscopes."

• Many cells involved in biologically relevant synapse formation, for example NK cells, are not adherent and, if forced to adhere, exhibit different behavior. Is this a limitation of the proposed method?

We thank the reviewer for this important comment. Indeed, our approach requires the adhesion of the cells to the cells. Thus, it was important to show in this study that the method does not disturb the physiological behavior of the cells. In order to check the physiological activity of the T cells in "adherent" mode we made the controls of the ability of these cells to get activated/kill on the coverslips (Fig. 1). Otherwise, we also showed in Fig. 2D-F imaging of adherent (melanoma) and non-adherent cells ($CD8^+$ T cells). Imaging interfaces between adherent cells through our method is of course much easier relative to imaging interfaces between non-adherent cells.

In summary, the method presented by the author seems versatile and robust, but even though many flavors of microscopy are applied to the created samples, the lack of quantitative details regarding the applied methods makes it hard to assess the results. Reporting quantitative parameters and settings is mandatory and will significantly improve the manuscript.

We thank again the reviewer for his encouraging and constructive comments, which we have now addressed point-by-point. We hope that these extensive revisions now allow the publication of our study.

Reviewers' comments:

Reviewer #1 (Remarks to the Author):

In the revised manuscript, the authors answered most of our questions and added more experimental data to support their claims. Now we are convinced that the technique reported in this manuscript may have some potential applications. However, we still have some more questions regarding the experimental details.
1.In figure 1D, the authors present the activated T cells by calcium imaging, and the killing efficiency was accessed by killing assay where ~40% of T2 cells were killed. During the imaging of the IS, how did the authors know which T cells were activated?
2.In figure 3D, the authors reported the observation of the co-clustering of CD80 and CD28. Is it possible to report the distances between these clusters? It is crucial to obtain relative positions between different molecules. In the SMLM, there is a problem in determining the relative positions between two colors. The authors may use multi-color fiducial markers to calibrate the shift in different colors due to chromatic aberration.
3.The authors claimed the observation of CD 45 clusters at IS in figure 4D. However, there are a lot of clusters in the cytosol (+1um) and on the membrance (+2um). Are they the same type of clusters or different? Can the authors elaborate more on this issue?
4.What is the resolution in live imaging in figure 4 (x, y, z, t)?

Reviewer #2 (Remarks to the Author):

Generally, I am happy to see that the authors provided additional details on the imaging and the experimental procedure in the revision of their manuscript. I think that this improved the understandability of the paper.

Nevertheless, I cannot recommend the revised manuscript for publication. The added information on the performed dSTORM experiments seem to me quite problematic. Visually, a prominent example is the striping of the membrane in the green channel in Figure S6. More important, however, are the highly unusual quality parameters reported (Figure S5). First, the authors state themselves that they frequently observed bimodal distributions. Indeed, most of the distributions are at least bimodal or exhibit significant shoulders, indicative of even more underlying unimodal distributions. For a fairly homogenous sample, this is quite surprising. Crucially, however, the reported values itself are concerning. The width of the fitted Gaussian (sigma) ranges sometimes up to more than 2 microns, but also down to less than 200 nm. This is hard to believe and, in fact, physically almost impossible, considering the reported imaging system. In addition, the offset of column D is reported to peak at 5000 and 10000 photons for the red and green channel, respectively, which is orders of magnitudes larger than for the other three cases. Surprisingly, this seemingly does not affect the reconstruction. Moreover, the most likely intensity of an individual signal seems to be almost zero for the red channel in column A, and the intensities of identical dyes vary by orders of magnitudes across different samples. Problematic as well are the reported distributions of localization uncertainties. They are similar for all conditions, even though this parameter should scale with the inverse of the square root of the detected photons, which cover a range from few hundreds to few 10000s of photons (by itself very unusual). For example, in column C, both the intensities in the red and green channel are reported to be very low, usually around 100-200 photons, but the localization precision is remarkably good, peaking at 10-20 nm. Compare this to column A, green channel, where the intensities go up to 15000 photons and the localization precision distribution is essentially the same.

If the authors insist on reporting dSTORM data, I am afraid that I can only ask them to essentially reanalyze/redo all related experiments and ensure proper recording and analysis of the single-molecule data. Furthermore, representative examples for raw data for all localization microscopy-

based experiments have to be provided as supplementary movies.

I am, however, not sure if such an effort is feasible within the scope of a second revision round. Perhaps it would be a wise choice to resubmit a modified manuscript where the focus is more on the method itself.

I do regret it that I cannot be more positive as the authors clearly invested a lot of work to revise the manuscript, but the shortcomings are just too substantial to ignore, especially since the dSTORM measurements are featured so prominently.

Reviewers' comments:

Reviewer #1 (Remarks to the Author):

In the revised manuscript, the authors answered most of our questions and added more experimental data to support their claims. Now we are convinced that the technique reported in this manuscript may have some potential applications. However, we still have some more questions regarding the experimental details.

We thank the reviewer for his comments and support.

1.In figure 1D, the authors present the activated T cells by calcium imaging, and the killing efficiency was accessed by killing assay where ~40% of T2 cells were killed. During the imaging of the IS, how did the authors know which T cells were activated?

We thank the reviewer for this comment and would like to clarify in response. In the calcium assay (Fig. 1D,E), we have used a fluorescent calcium indicator (Fluo-4). Thus, we have recognized activated cells via their elevation of green fluorescence. In the separate killing assay (Fig. 1F) we counted cells that were alive or dead. Killed cells were identified by Trypan Blue staining. We now clarify in the text that the $Ca^{++}$ and killing assays are from separate experiments, and refer the reader to the Methods for further details.

2.In figure 3D, the authors reported the observation of the co-clustering of CD80 and CD28. Is it possible to report the distances between these clusters? It is crucial to obtain relative positions between different molecules. In the SMLM, there is a problem in determining the relative positions between two colors. The authors may use multi-color fiducial markers to calibrate the shift in different colors due to chromatic aberration.

In our imaging assay, registration between two spectral channels is typically very accurate since we use multiband (quad-) dichroic mirror that doesn't move during acquisition of the two channels. This overcomes most registration issues (which may be more common in other imaging systems). As the reviewer suggests, registration was demonstrated by imaging 100 nm Tetraspeck beads at the relevant spectral channels (as shown below; scale bar – 500nm). As for CD80 and CD28 in (now) Fig. 3C – the clusters of these molecules seem to overlap and there doesn't seem to be a clear separation distance between them.

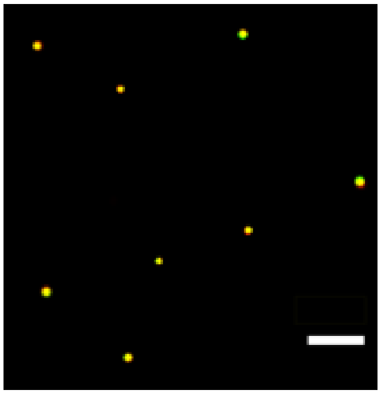

3.The authors claimed the observation of CD45 clusters at IS in figure 4D. However, there are a lot of clusters in the cytosol (+1um) and on the membrane (+2um). Are they the same type of clusters or different? Can the authors elaborate more on this issue?

We thank the reviewer for raising this point. To clarify - CD45 is a membrane protein, which has been stained in our experiments on the surface of the cells. Specifically, the cell in (now) Fig. 2E seems to be ruffled. Thus, its 2D cross-sections in planes +1 and +2 contain CD45 clusters at the ruffled membrane. We now further clarify this point in the text, as follows:

"We note that our staining of CD45 was limited to the surface of the cells. Thus, CD45 appearance in 2D cross-sections in planes +1 and +2 in Fig. 2D is likely due to the ruffled T cell membrane (also evident in Fig. 2E)."

4.What is the resolution in live imaging in figure 4 (x, y, z, t)?

We would like to clarify regarding the live-cell resolution in our study. The axial resolution of SMLM reconstruction of live cell imaging is detailed in Fig S5. It often peaks at 20-30nm. The resolution along the Z axis is diffraction-limited, as we haven't attempted to improve it in the current study. The temporal resolution has not been explored in this study using SMLM. Still, we have demonstrated in recent publications 2-color SMLM images in live cells with temporal resolution down to 3-5 sec [Razvag et al. Nature Communications 9, 732 (2018) ]. Faster (over video-rate) imaging may be obtained using confocal imaging. Notably, our approach also increases the lateral resolution for such diffraction-limited imaging of cell-cell interfaces.

Reviewer #2 (Remarks to the Author):

Generally, I am happy to see that the authors provided additional details on the imaging and the experimental procedure in the revision of their manuscript. I think that this improved the understandability of the paper.

We thank the reviewer for recognizing our major revisions and efforts for improvement of the manuscript in the previous revision cycle.

Nevertheless, I cannot recommend the revised manuscript for publication. The added information on the performed dSTORM experiments seem to me quite problematic.

We thank the reviewer for his detailed review of our revisions. In response to the new comments, we have now critically compared the performance of SMLM imaging using our approach (imaging cells-on-cells) to the 'gold-standard' of imaging in total internal reflection microscopy (TIRF). We show that the performance using these approaches is rather similar, and discuss specific differences where exist. All of this is detailed below in response to the individual comments.

Visually, a prominent example is the striping of the membrane in the green channel in Figure S6.

The reviewer points to an artifact in dSTORM rendering that is typical to relatively low signals. In such signals, the structure of the detector pixels becomes visible. We appreciated this comment, and have now replaced the image in Fig. S6. The stronger signal eliminates the striping effect that was visible before. We have not noticed such striping in other images.

Figure S6

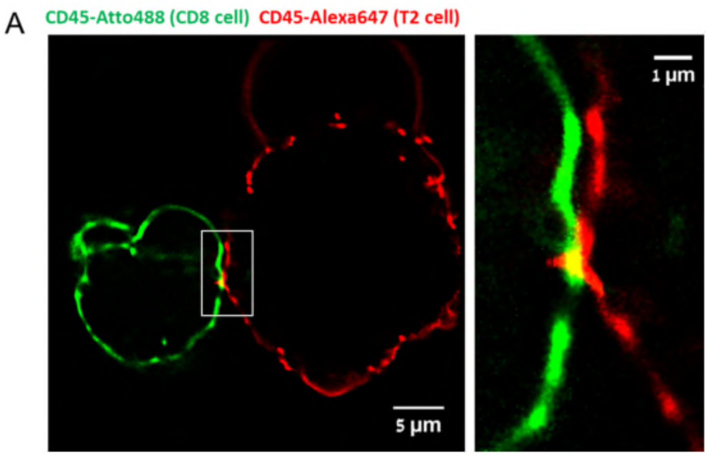


More important, however, are the highly unusual quality parameters reported (Figure S5). First, the authors state themselves that they frequently observed bimodal distributions. Indeed, most of the distributions are at least bimodal or exhibit significant shoulders, indicative of even more underlying unimodal distributions. For a fairly homogenous sample, this is quite surprising.

We thank the reviewer for his important comment. We would like to clarify regarding the apparent bimodality in some of our histograms of localization parameters:

1. Bimodality is fluorophore specific. Some fluorophores blink better than others and are more suitable for dSTORM imaging. A common example of such a favorable fluorophore is Alexa647. Some fluorophores blink to a lower extent (e.g. Atto488), but have been employed for 2 color dSTORM [Dempsey et al, Nat Methods. 2011 Dec; 8(12): 1027–1036]. We have further found that DiD did not blink well, and thus have removed it from our dSTORM imaging.

2. Following the reviewer's comment, we have now studied closer the cause for the bimodality. It seems that our samples demonstrate inhomogeneous patterning molecules and background: First, our samples include the bottom and the upper cells. The bottom cell often creates a non-homogeneous background field when imaging the upper cell surface. This increased background is more dominant for fluorophores with lower extent of blinking. Thus, we now recommend that labeling of these bottom cells would be with the fluorophores that blink more optimally.
Second, molecules may organize in both monomers and aggregates in the same cell. Imaging of aggregates may result in extended localizations with a relatively high intensity, and consequently – (artificially) low uncertainty. This can be seen (as an example below) for Atto488. We detail below our rectification of such issues.

Crucially, however, the reported values itself are concerning.

We thank the reviewer for this comment. In response, we have now conducted a detailed comparison of our imaging of cell-cell conjugates with our imaging of the **same fluorophores** in cells adherent to coverslips and captured in total internal reflection microscopy (**TIRF**). TIRF imaging limits the acquired data to <200nm above the coverslip and thus reduces most sources of background.
Importantly we find skewness in the histograms of localization parameters in the TIRF images. Notably, the histograms of our cell-on-cell images are not dramatically different than those of the TIRF images. Differences in the histograms mainly appear for the offset parameter of Atto488 (which we assign above mainly to the lower cell background). Thus, we conclude that our cell-on-cell imaging technique has not abrogated dSTORM imaging much beyond TIRF imaging.

These results now appear in a modified Fig. S5.

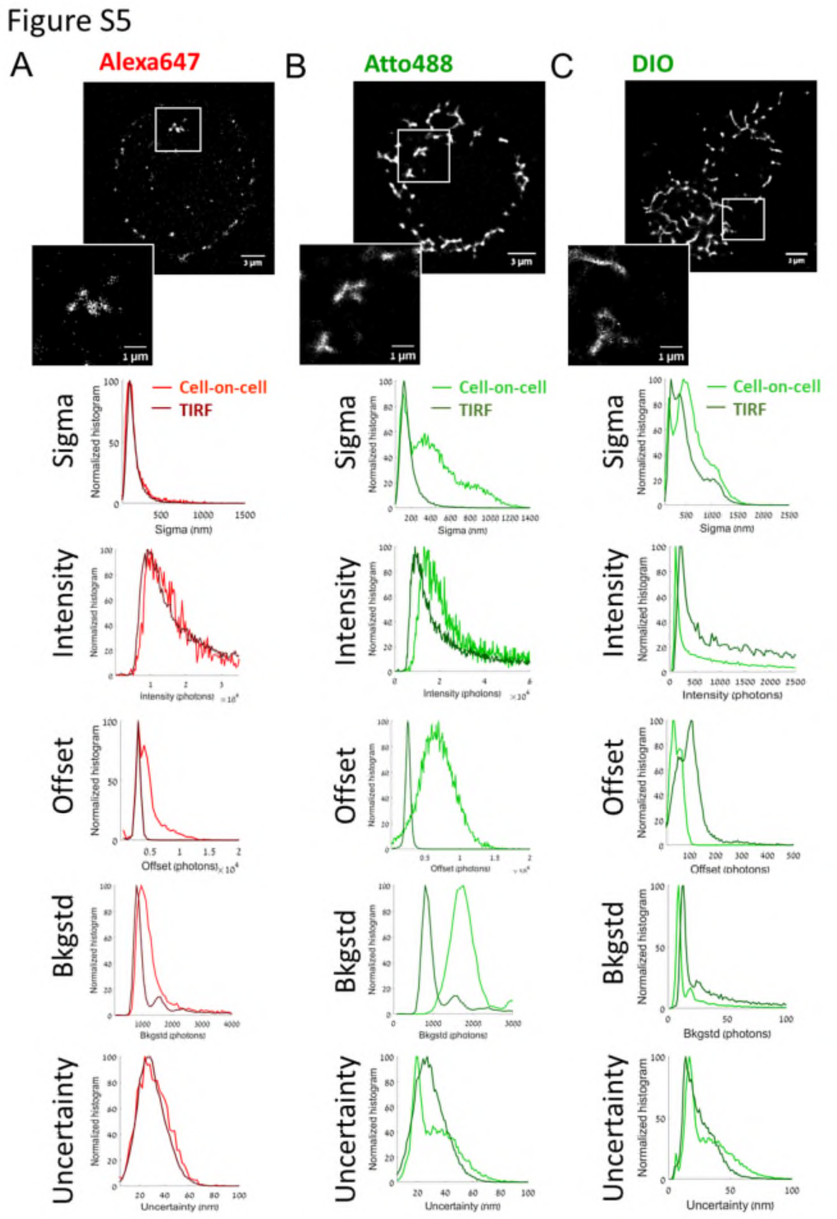


The width of the fitted Gaussian (sigma) ranges sometimes up to more than 2 microns, but also down to less than 200 nm. This is hard to believe and, in fact, physically almost impossible, considering the reported imaging system.

We agree with the reviewer that the larger sigma values are problematic. We studied this in cells stained by Atto488 (see analyses in the presented panels below). We observe that localizations with relatively large sigma values (>650) are enriched at the centers of aggregates that are misidentified as single molecules (filtered localizations are shown in magenta and all localizations, i.e. without filtering, are shown in blue). We now filtered these localizations for Atto488 and for DiO. Localizations with sigma that peaked around 200nm were kept as they appear also in TIRF imaging (Updated Fig. S5).

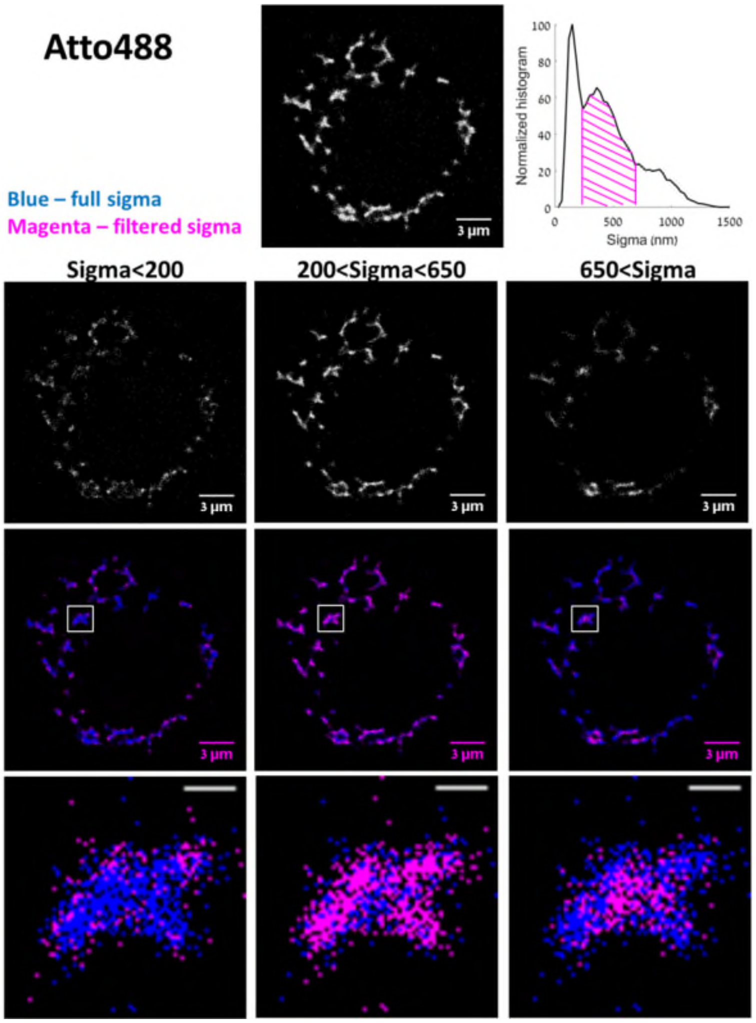


In addition, the offset of column D is reported to peak at 5000 and 10000 photons for the red and green channel, respectively, which is orders of magnitudes larger than for the other three cases. Surprisingly, this seemingly does not affect the reconstruction.

We note by TIRF that the offset for Alexa647 is ~3,000 photons and is rather similar to our cell-on-cell imaging. For Atto488, our cell-on-cell imaging results in offset peak at ~7,500 photons and is significantly larger than for TIRF. This offset is due to the increased background (in this case from the cytosolic volume of the upper cell, which has been labeled with Atto488).

Moreover, the most likely intensity of an individual signal seems to be almost zero for the red channel in column A, and the intensities of identical dyes vary by orders of magnitudes across different samples.

The intensity of all fluorophores are now compared between TIRF and cell-on-cell. The results (new Fig. S5) show rather similar intensity distributions for all fluorophores between these two imaging modalities.

Problematic as well are the reported distributions of localization uncertainties. They are similar for all conditions, even though this parameter should scale with the inverse of the square root of the detected photons, which cover a range from few hundreds to few 10000s of photons (by itself very unusual). For example, in column C, both the intensities in the red and green channel are reported to be very low, usually around 100-200 photons, but the localization precision is remarkably good, peaking at 10-20 nm. Compare this to column A, green channel, where the intensities go up to 15000 photons and the localization precision distribution is essentially the same.

Following the reviewer's comment, we have now checked the localization uncertainty distributions. The higher-intensity part of the intensity histograms is related to the lower part of the uncertainty histograms. Specifically, we have removed the data for DiD, and our results now show overall similar results between cell-on-cell imaging and TIRF imaging of cells on coverslips (see again in new Fig. S5).

If the authors insist on reporting dSTORM data, I am afraid that I can only ask them to essentially reanalyze/redo all related experiments and ensure proper recording and analysis of the single-molecule data. Furthermore, representative examples for raw data for all localization microscopy-based experiments have to be provided as supplementary movies.

Following the reviewer's comment, we have now revisited and re-analyzed all images that we have found problematic in terms of localization parameters. First, we have removed the data with DiD staining for dSTROM imaging. Second, we have filtered out localizations of Atto488 and of DiO that had relatively high Sigma values (>650nm) as they originated from molecular aggregates. Modified figures are: 3 A-D, 4A, S5, S6 A-B.

We also provide movies of cells captured via our cell-on-cell approach. For clarity, the movies are shown separately for our used fluorophores in this study, namely - Alexa647, Atto488, DiO and Dronpa.

I am, however, not sure if such an effort is feasible within the scope of a second revision round. Perhaps it would be a wise choice to resubmit a modified manuscript where the focus is more on the method itself.

We thank the editor and the reviewer for the opportunity to address the raised concerns within a second revision of our manuscript.

I do regret it that I cannot be more positive as the authors clearly invested a lot of work to revise the manuscript, but the shortcomings are just too substantial to ignore, especially since the dSTORM measurements are featured so prominently.

We thank the reviewer for his detailed comments regarding our SMLM imaging. Following these comments, we have now reanalyzed our data. We show that the large extent of our imaging is comparable in quality (i.e. localization parameters) to the 'gold-standard' of SMLM imaging in TIRF mode. We also identify specific cases of fluorophores and molecular targets that require caution, and recommend ways to avoid and improve possible related issues. We have further reanalyzed the dSTORM data to avoid such issues.
We feel that these modifications to the manuscript have significantly improved it, and we hope that it is now acceptable for publication.

REVIEWERS' COMMENTS:

Reviewer #1 (Remarks to the Author):

The authors answered all of our questions. We have no more questions.

Reviewer #2 (Remarks to the Author):

The authors have addressed my previous comments in detail. I would like to thank them for their remarkable effort, which considerably improved the paper. I am happy to recommend the manuscript for publication in its current form.
